# NIR-excitable heterostructured upconversion perovskite nanodots with improved stability

**DOI:** 10.1038/s41467-020-20551-z

**Published:** 2021-01-11

**Authors:** Longfei Ruan, Yong Zhang

**Affiliations:** 1 Department of Biomedical Engineering, Faculty of Engineering, National University of Singapore, Singapore, 117583 Singapore; 2 NUS Graduate School for Integrative Sciences and Engineering, National University of Singapore, Singapore, 117456 Singapore

**Keywords:** Nanoparticles, Quantum dots

## Abstract

There is a great need to develop heterostructured nanocrystals which combine two or more different materials into single nanoparticles with combined advantages. Lead halide perovskite quantum dots (QDs) have attracted much attention due to their excellent optical properties but their biological applications have not been much explored due to their poor stability and short penetration depth of the UV excitation light in tissues. Combining perovskite QDs with upconversion nanoparticles (UCNP) to form hybrid nanocrystals that are stable, NIR excitable and emission tunable is important, however, this is challenging because hexagonal phase UCNP can not be epitaxially grown on cubic phase perovskite QDs directly or vice versa. In this work, one-pot synthesis of perovskite-UCNP hybrid nanocrystals consisting of cubic phase perovskite QDs and hexagonal phase UCNP is reported, to form a watermelon-like heterostructure using cubic phase UCNP as an intermediate transition phase. The nanocrystals are NIR-excitable with much improved stability.

## Abstract (web summary)

Combining two or more different materials with different crystal structures into single nanoparticle with combined advantages is challenging. Here, the authors demonstrate one-pot synthesis of perovskite-upconversion hybrid nanocrystals consisting of cubic and hexagonal phases to form a watermelon-like heterostructure.

## Introduction

There is increasing interest in developing heterostructured nanocrystals that combine two or more different materials into single nanoparticles with combined advantages. For example, heterostructured quantum dots (QDs) such as InP/ZnS, CdSe/CdS/ZnS, Si/CdS, PbSe/PbS, and CdSe/CdTe QDs have been synthesized and have exhibited significantly improved optical and electrical properties as compared to their individual constituents^1,2^. QDs are semiconductor nanoparticles whose excitons are limited to the Bohr radius in three-dimensional space, showing the quantum confinement effect^3^. Traditional quantum dots are made of compounds of the IV, II−VI, IV−VI, III−V, and I−III−VI groups, such as Si, CdTe, CdSe, ZnO, PbS, InP, CuInS_2_ QDs, etc^4,5^. In recent years, lead halide perovskite QDs have attracted much attention due to their excellent optical properties such as high photoluminescence quantum yield (PLQY) up to 100%^6–8^, large UV absorption cross section^9^, tunable emission over the entire visible spectrum^10,11^, long carrier diffusion length, and high carrier mobility^12^, which make them suitable for use in high-performance solar cells, light-emitting diodes (LEDs), lasers, and photodetectors^10–14^. However, perovskites have poor stability which limits their applications. Furthermore, their use in biological and medical applications is also very limited due to the low penetration depth of UV light in biological tissues. As compared to perovskite QDs, lanthanide-doped upconversion nanocrystals (UCNP) such as NaYF_4_:Yb,Tm have good stability and much higher multi-photon absorption efficiency under NIR excitation using low-cost continuous-wave (CW) lasers^15,16^. UCNP have been used in deep tissue fluorescence imaging and phototherapy due to deep penetration of NIR light in biological tissues^17–20^. However, UCNP have fluorescence emissions at fixed wavelengths only and their emission colors are not tunable and as such their use in multiplexed bioimaging or bioassays is limited. Combining perovskite QDs with UCNP to form hybrid nanocrystals with a heterostructure that are stable, NIR excitable and emission tunable may address the above-mentioned issues. Some studies have been performed to synthesize hybrid perovskite nanocrystals with semiconductor quantum dots with small crystal phase-mismatch such as CsPbBr_3_-CdS^21^, CsPbI_3_-PbS^22^, and CsPbBr_3_-TiO_2_^23^ and bulk materials, such as MAPbI_3_-PbS^24^ and CsPbX_3_-PbS (X = Cl, Br, and I)^25^. Previous attempts have been made to synthesize UCNP-perovskite nanohybrids. The synthesis of NaYF_4_:Yb,Tm@CH_3_NH_3_PbBr_3_ nanohybrids via an innovative strategy consisting of using cucurbituril to anchor the perovskite nanoparticles firmly and closely to the upconversion nanoparticles has been previously achieved^26^. This is a typical A + B synthesis strategy. Material A and B were first synthesized, and an intermediate molecule cucurbituril was used to link the two materials to obtain AB composite material. The composite of perovskite NWs and UCNPs was synthesized at room temperature using PVP as a template^27^. First, the PVP-modified UCNPs were synthesized, and they were mixed with Pb(OA)_2_, which was a perovskite NWs precursor. l-cysteine was added and dispersed in isopropanol solution, and CH_3_NH_3_Br, another precursor of NWs, was added to obtain an AB composite. It is typical to synthesize material A first and then rely on the template method to get the composite material of AB during the synthesis process of material B. They all rely on the action of chemical bonds to connect the two synthetic materials, while we have creatively introduced the intermediate synthesis of crystal phase by controlling the crystal growth, and use the phase-transition characteristics to obtain heterojunction materials with excellent optical properties. However, it remains a challenge to synthesize perovskite/UCNP heterostructured nanocrystals due to the structural difference between cubic-phase perovskite QDs and hexagonal-phase UCNP and lack of a good understanding of the growth/formation mechanism. Going beyond perovskites and UCNP, this has posed a challenge in synthesizing heterostructured nanocrystals consisting of two or more different crystal phases with structural and lattice mismatch and a generic strategy must be developed.

In general, some stringent requirements must be met for making heterostructured nanocrystals: (I) crystal structure matching. Cubic-phase CsPbBr_3_ crystals can grow together with cubic-phase CdS or PbS crystals^21,25^; (II) lattice parameter matching. MAPbI_3_–PbS lattice mismatch was as small as 4.9%^24^; (III) similar reaction temperature and condition^21^. There are two strategies for synthesizing A–B hybrid nanocrystals with two different crystals A and B: (I) monodispersed A nanocrystals are synthesized first and then B is epitaxially grown on the surface of A nanocrystals to form a core-shell structure. (II) A and B are synthesized in the same solution simultaneously to produce hybrid nanocrystals with A embedded in B or B embedded in A. Both strategies require A and B to have the same crystal structure and closely matched structural and lattice parameters. For synthesizing perovskite–UCNP hybrid nanocrystals, in this work CsPbBr_3_-NaYF_4_:Yb,Tm, strategy II is adopted. Multiple CsPbBr_3_ perovskite QDs are embedded in the NaYF_4_:Yb,Tm UCNP to ensure there is sufficient absorption of NIR excitation light and efficient energy transfer from the UCNP to perovskite QDs. On the other side, it has been widely reported that CsPbBr_3_ perovskite QDs have very poor stability in water^23,28^. Embedding them in the UCNP will help prevent them from dissolving in water. The challenge to overcome is how to embed cubic-phase CsPbBr_3_ QDs in hexagonal-phase NaYF_4_:Yb,Tm UCNP to form hybrid nanocrystals in the same synthetic solution by one-pot synthesis. It is noted that the lattice mismatch between cubic-phase CsPbBr_3_ QDs and NaYF_4_:Yb,Tm UCNP is small, 5.9% only. Our hypothesis is those hybrid nanocrystals consisting of cubic-phase CsPbBr_3_ QDs and cubic-phase NaYF_4_:Yb,Tm UCNP can be first prepared in one-pot based on co-precipitation method due to small crystal structure mismatch and similarity in synthetic conditions. Phase transition of the UCNP from cubic phase to hexagonal phase is then induced by heating to a higher temperature, resulting in the formation of hybrid nanocrystals with cubic phase CsPbBr_3_ QDs embedded in hexagonal-phase NaYF_4_:Yb,Tm UCNP^29^.

In this work, we have demonstrated the successful synthesis of monodispersed and oval-shaped watermelon-like hybrid nanocrystals consisting of small cubic-phase CsPbBr_3_ QDs (watermelon seeds) embedded in hexagonal-phase NaYF_4_:Yb,Tm UCNP (watermelon pulp) with a thin NaYF_4_:Yb,Tm shell (watermelon skin). The hybrid nanocrystals emit characteristic green fluorescence of the CsPbBr_3_ QDs upon UV excitation and UV-blue fluorescence of the NaYF_4_:Yb,Tm UCNP upon NIR excitation, demonstrating the co-existence of both CsPbBr_3_ phase and NaYF_4_:Yb,Tm phase in the same structure, and green fluorescence upon NIR excitation when the NaYF_4_:Yb,Tm phase absorbs NIR light and transfers the energy to the CsPbBr_3_ phase. This has opened a new way of synthesizing heterostructured nanocrystals with two distinctly different crystal phases via an intermediate transition phase and the strategy could be applied to many other materials.

## Results

### Synthesis of CsPbBr_3_ QDs and NaYF_4_:Yb,Tm UCNP

CsPbBr_3_ QDs with regular cubic shape were first synthesized based on Kovalenko’s method^30^. The size of the CsPbBr_3_ QDs was 12.6 nm, and the diffraction peaks at 2*θ* = 15.2, 21.6, 26.5 30.6, 34.4, 37.8, 43.9, 46.7, 49.4, 54.5, and 59.3° correspond to diffractions from the {100}, {110}, {111}, {200}, {210}, {211}, {220}, {300}, {310}, {222}, and {321} planes of the cubic-phase CsPbBr_3_ (PDF# 00-054-0752) (Supplementary Figs. 1 and  2). To analyze the optical properties, the UV−vis absorption spectra, and the PL emission and excitation spectra (Supplementary Fig. 3) was obtained from the colloidal solution of the CsPbBr_3_ nanocrystals. The PL emission spectrum showed a peak position at 515 nm and showed a strong green fluorescence under 365 nm excitation (Supplementary Fig. 3A). The absorption onset of CsPbBr_3_ nanocrystals was around 510 nm (Supplementary Fig. 3B). The time-resolved PL decay of CsPbBr_3_ showed average PL decay lifetimes was 18.4 ns (Supplementary Fig. 3C and Supplementary Table 1). NaYF_4_:30%Yb,0.5%Tm nanocrystals were synthesized by the high-temperature co-precipitation method^31^. When the temperature reached 300 °C, NaYF_4_:30%Yb and 0.5%Tm nanocrystals with a size of about 5 nm were synthesized, and they emitted a weak fluorescence under 980 nm excitation (Supplementary Fig. 5). At 300 °C for 60 min, uniform NaYF_4_:30%Yb,0.5%Tm nanocrystals with a size of 32.5 nm were synthesized, and they emitted a strong blue fluorescence under 980 nm excitation (Supplementary Fig. 6). According to the XRD results (Supplementary Fig. 7), the diffraction peaks at 2*θ* = 17.2, 30.1, 30.8, 34.6, 39.6, 43.5, 46.5, 52.1, 53.1, 53.7, and 55.2° correspond to diffractions from the {100}, {110}, {101}, {200}, {111}, {201}, {210}, {002}, {300}, {211}, and {102} planes of the hexagonal-phase NaYF_4_ (JCPDS: 00-016-0334).

### Synthesis of heterostructured nanocrystals

**Fig. 1:**
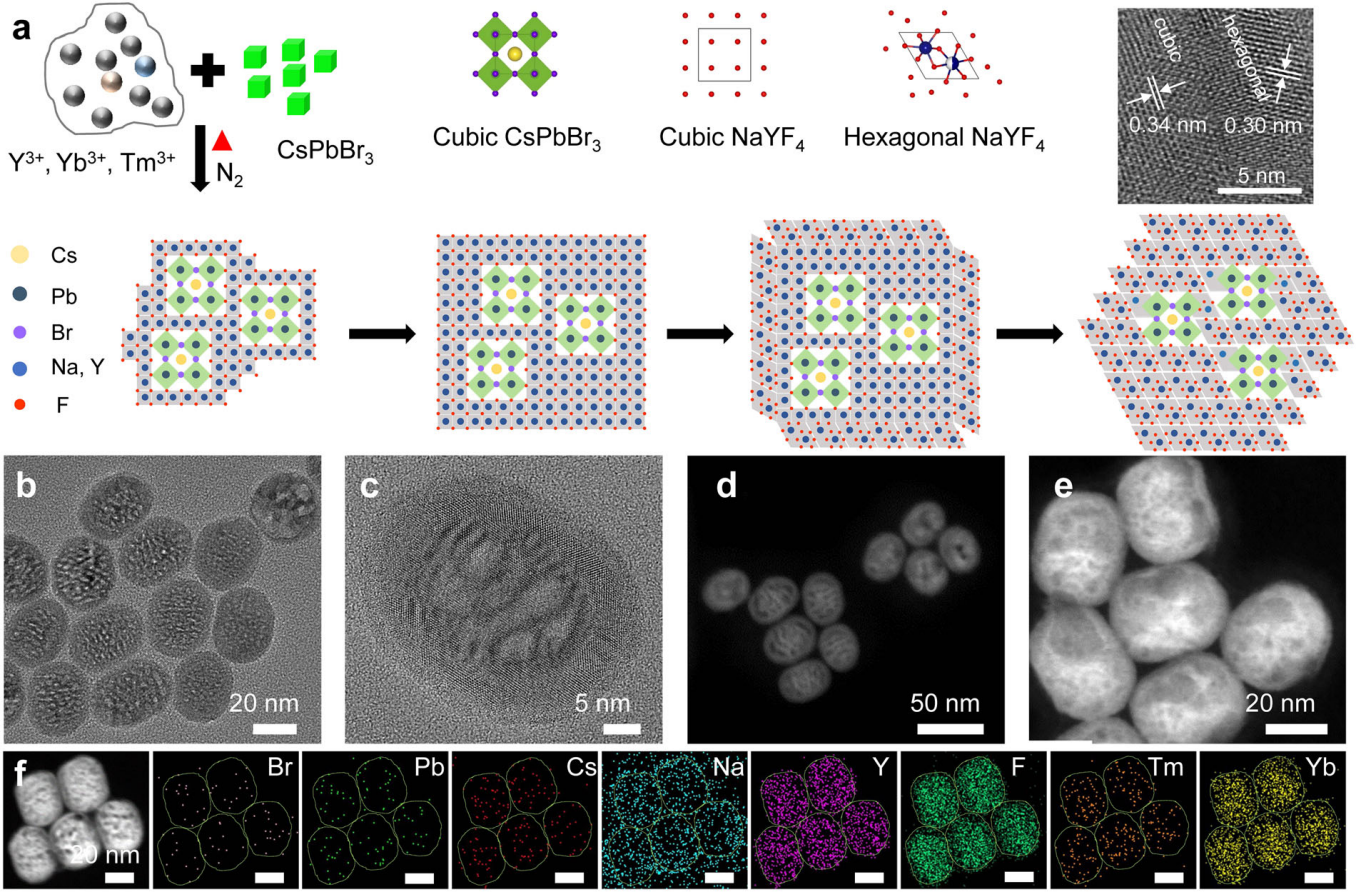
Synthesis of CsPbBr_3_-NaYF_4_:Yb,Tm hybrid nanocrystals. **a** Schematic showing the formation of heterostructured CsPbBr_3_–NaYF_4_: Yb,Tm nanocrystals, **b**, **c** TEM and **d**, **e** STEM images of heterostructured CsPbBr_3_–NaYF_4_:Yb,Tm nanocrystals. **f** Elemental mapping of heterostructured CsPbBr_3_–NaYF_4_:Yb,Tm nanocrystals. Scale bars in **f**, 20 nm.

As shown in Fig. 1a, cubic-phase NaYF_4_:Yb,Tm UCNP can be epitaxially grown on the surface of cubic-phase CsPbBr_3_ QDs due to the similarity in the crystal structure and lattice. When the temperature is increased, UCNP is converted from cubic to hexagonal phase, while cubic-phase CsPbBr_3_ QDs remain unchanged so hexagonal-phase UCNP is formed on the surface of cubic-phase CsPbBr_3_ QDs. CsPbBr_3_-NaYF_4_:Yb,Tm hybrid nanocrystals were synthesized using a high-temperature co-precipitation method. Their formation and crystal structures were studied. CsPbBr_3_ QDs with regular cubic shape were first synthesized based on Kovalenko’s method (Supplementary Fig. 1)^11^, and then added to the precursor solution for UCNP synthesis which consists of various lanthanide ions such as Y^3+^, Yb^3+^, and Tm^3+^ and surfactants, such as oleic acid (OA) and octadecylene (ODE). As shown in Fig. 1b–e, heterostructured nanocrystals were formed with the CsPbBr_3_ QDs embedded in the NaYF_4_:Yb,Tm UCNP, to form a structure similar to that of watermelon with multiple seeds. The average size of the nanocrystals was ~37 nm, and the outer shell was ~3.7-nm thick (Fig. 1c). CsPbBr_3_ QDs were found to be in the middle region of the nanocrystals with distinct crystal boundary formed between the CsPbBr_3_ QDs and UCNP from the bright- and dark-field TEM images. The heterostructure interface can be clearly seen from the HRTEM diagram (Supplementary Fig. 8), and the area segmented by the arc presents obvious light and dark patterns. Above the arc is the cubic-phase CsPbBr_3_ crystals and below the arc is the hexagonal-phase NaYF_4_:Yb,Tm crystals. The cubic-phase NaYF_4_:Yb,Tm is a metastable crystalline phase, which led to the formation of irregular small cubic-phase CsPbBr_3_ crystals within the heterogeneous junction, thus making the interface of heterogeneous junction irregular. From HRTEM, STEM, and elemental imaging results (Supplementary Fig. 9), it can be clearly seen that the perovskites in the heterostructured nanocrystals were small irregular particles with a size of 6–12 nm. At 250 °C, the heterostructured nanocrystals were composed of a cubic-phase UCNPs and cubic-phase perovskites. The cubic-phase UCNPs has a high-temperature metastable crystalline phase, which led to the irregular growth of perovskites in the heterojunction. Subsequently, under the effects of thermodynamics (250–300 °C), the cubic-phase UCNP transited to the hexagonal phase. At this time, the epitaxial growth was mainly due to the hexagonal-phase UCNP, and most of the perovskite stopped growing due to the mismatch of crystal phases. As shown in Fig. 1f, eight elements including Br, Pb, Cs, Na, Y, F, Tm, and Yb were distributed in the nanocrystal structure (Supplementary Fig. 10). The elemental mapping images showed that Na, Y, F, Tm, and Yb ions which constitute the NaYF_4_:Yb,Tm UCNP were uniformly distributed throughout the hybrid nanocrystals, while Br, Pb, and Cs ions which make up CsPbBr_3_ QDs were located in the middle part. This could also be seen from the images of single-particle element imaging (Supplementary Fig. 11), supporting that the hybrid nanocrystals were formed with the CsPbBr_3_ QDs embedded in the NaYF_4_:Yb,Tm UCNP. According to the XPS results, the concentration ratio of CsPbBr_3_ and NaYF_4_:Yb,Tm in the heterostructured nanocrystals was 1:59. (Supplementary Table 2).

### Nanocrystal heterostructure formation during heat treatment

**Fig. 2:**
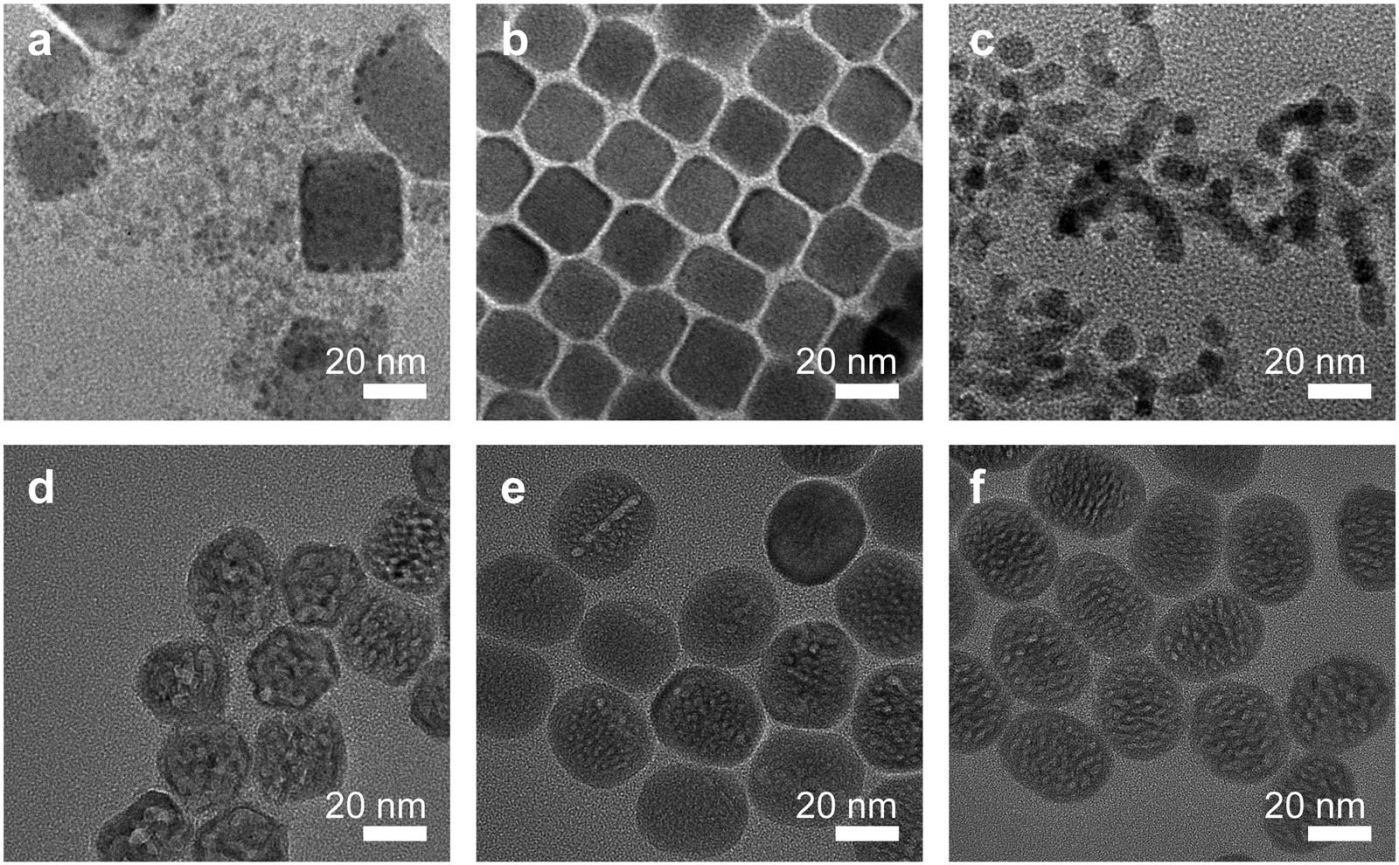
Nanocrystal heterostructure formation during heat treatment. TEM images of heterostructured CsPbBr_3_–NaYF_4_: Yb,Tm nanocrystals collected during the synthesis at different temperatures. **a** 150 °C, **b** 200 °C, **c** 250 °C, **d** 300 °C—0 min, **e** 300 °C-30 min, **f** 300 °C—60 min.

The formation of the CsPbBr_3_-NaYF_4_:Yb,Tm hybrid nanocrystals was studied by TEM measurement of the samples collected at different temperatures during the heat-treatment process. From the literature, it was reported that cubic-phase CsPbBr_3_ crystals (5.83 Å) and cubic-phase NaYF_4_ crystals (5.47 Å) have a small lattice mismatch of ~5.9%, suggesting that theoretically it is possible to grow cubic phase CsPbBr_3_ crystals on cubic-phase NaYF_4_ crystals epitaxially or vice versa^32–34^. In this study, pre-made cubic-phase CsPbBr_3_ QDs were added to the precursor solution to synthesize NaYF_4_:Yb,Tm UCNP, so it is a high probability that NaYF_4_:Yb,Tm crystals were grown on the surface of CsPbBr_3_ QDs, evidenced from the dark spots formed on the surface of the cubic-shape nanocrystals at 150 °C and 200 °C, as shown in Fig. 2a, b. This is similar to cubic-phase CsPbX_3_–PbS hybrid nanocrystals formed under a similar condition^25^. The temperature was subsequently increased to 250 °C, resulting in much smaller nanocrystals with a size of ~12 nm (Fig. 2c). Besides poor chemical stability in water, poor thermostability of CsPbBr_3_ QDs has also been well reported^35,36^. High temperature may cause the oxidation and hydration and eventually the decomposition of CsPbBr_3_ QDs. At 250 °C, the size of the nanocrystals was apparently much smaller as compared to a lower temperature, suggesting that CsPbBr_3_ QDs started to decompose partially. This is usually considered a problem for perovskite QDs; however, in this study, the smaller size of perovskite QDs caused by partial decomposition makes it possible to synthesize small perovskite–UCNP hybrid nanocrystals. On the other side, the cubic phase is dominant in the NaYF_4_ nanocrystals formed at 250 °C, as previously reported^29,37,38^. The small hybrid nanocrystals served as the seeds for further growth of the NaYF_4_:Yb,Tm UCNP. The average size of the nanocrystals increased to 30.6 nm when the temperature reached 300 °C (Fig. 2d). With the extension of the insulation time to 30 and 60 min, the size continued to increase to 34.5 nm and 37.1 nm, respectively (Fig. 2e, f). Their morphology changed from square or hexagonal to ellipsoidal shape. In addition, multiple cores were clearly observed in the middle region of the perovskite–UCNP hybrid nanocrystals during the thermal insulation process (Supplementary Fig. 12), which was consistent with the previous elemental mapping results.

### Luminescence emission profile

**Fig. 3:**
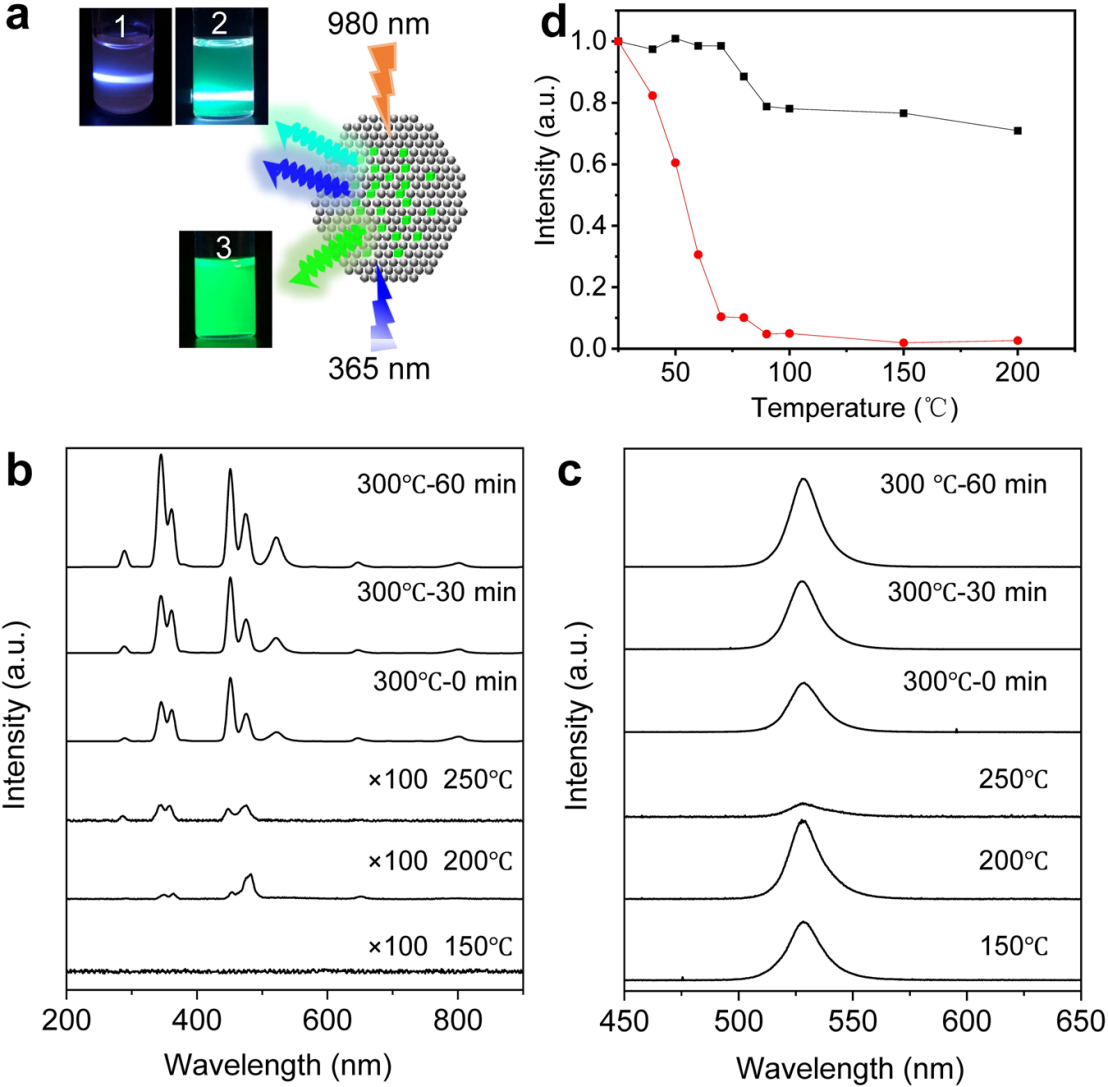
Fluorescence of CsPbBr_3_–NaYF_4_:Yb,Tm hybrid nanocrystals. **a** Photographs showing different color fluorescence emitted from the heterostructured CsPbBr_3_-NaYF_4_:Yb,Tm nanocrystals collected during the synthesis under different conditions (state 1, 300 °C—0 min, state 2, 300 °C—60 min, both under 980 nm excitation, state 3, 300 °C—60 min, under 365 nm excitation). **b**, **c** Fluorescence emission spectra of heterostructured CsPbBr_3_–NaYF_4_:Yb,Tm nanocrystals at different temperatures under 980 nm and 365 nm excitations, respectively. **d** Fluorescence intensity of CsPbBr_3_ nanocrystals (red) and heterostructured CsPbBr_3_–NaYF_4_:Yb,Tm nanocrystals (black) collected at different temperatures under 365 nm excitation.

The fluorescence emission of nanocrystals is dependent on the crystal structure and phases. Fluorescence emission spectra of the perovskite–UCNP hybrid nanocrystals collected at different temperatures were measured under excitation at 980 nm and 365 nm, respectively, and used to study the crystal phase formation and transition during the synthesis of the hybrid nanocrystals. As shown in Fig. 3a, the hybrid nanocrystals had three different states of luminescence: it emitted blue and cyan fluorescence under 980 nm excitation and green fluorescence under 365 nm excitation. When the temperature was increased from 150 °C to 250 °C, upconversion luminescence was weak and there was no characteristic green fluorescence emission of CsPbBr_3_ QDs at 515 nm under 980 nm excitation, indicating that there is no energy transfer from the NaYF_4_:Yb,Tm UCNP to CsPbBr_3_ QDs due to the low luminescence efficiency of cubic-phase NaYF_4_:Yb,Tm UCNP formed at low temperature^38,39^. The intensity of the green fluorescence of CsPbBr_3_ QDs under 365 nm excitation decreased due to the partial decomposition of the QDs. When the temperature was further increased to 300 °C, the hybrid nanocrystals exhibited UV/blue upconversion fluorescence under 980 nm excitation (Fig. 3b), with emission peaks located at 292 nm (^1^I_6_ → ^3^F_4_), 347 nm (^1^I_6_ → ^3^F_4_), 362 nm (^1^D_2_ → ^3^H_6_), 450 nm (^1^D_2_ → ^3^F_4_), and 478 nm (^1^G_4_ → ^3^H_6_), and green fluorescence at 522 nm corresponding to the emission of CsPbBr_3_ QDs, suggesting a significant energy transfer from the NaYF_4_:Yb,Tm UCNP to CsPbBr_3_ QDs and formation of hexagonal-phase NaYF_4_:Yb,Tm UCNP. It has been reported that hexagonal-phase NaYF_4_:Yb,Tm UCNP emits much stronger upconversion fluorescence as compared to its cubic phase^38–40^. During the process of maintaining the temperature at 300 °C for 0 to 60 min, the intensity of the green fluorescence emission peak increased, suggesting an increased energy transfer from the NaYF_4_:Yb,Tm UCNP to CsPbBr_3_ QDs. Meanwhile, at 300 °C, the hybrid nanocrystals emitted strong green fluorescence at 525 nm under 365 nm excitation which was corresponding to CsPbBr_3_ QDs (Fig. 3c). The absorption of the heterostructure composite of CsPbBr_3_–NaYF_4_:Yb,Tm nanocrystals were around 510 nm. The excitation spectrum showed that the heterostructure composite of CsPbBr_3_–NaYF_4_:Yb,Tm nanocrystals exhibited the strongest peak at 475 nm monitored at the emission wavelength of 525 nm, which is in good agreement with the result from the UV–vis absorption spectrum (Supplementary Fig. 13). During the process of nanocrystals formation and growth, the heterostructured nanocrystal at 250 °C was composed of cubic-phase UCNPs and cubic-phase CsPbBr_3_. When the temperature increased to 300 °C and incubated for 60 min, the cubic-phase UCNPs changes to the hexagonal phase and resulted in the heterostructured nanocrystals of the hexagonal-phase UCNPs and cubic-phase CsPbBr_3_ formation. The PLQY of these heterostructured nanocrystals under excitation at 980 nm were as follows: 150 °C (0%), 200 °C (0%), 250 °C (0.02%), 300 °C—0 min (0.15%), and 300 °C—60 min (0.25%) (Supplementary Table 3). Similarly, the PLQY of these heterostructured nanocrystals increased from 0.02% to 0.139% under the 980 nm excitation. This PLQY increase was a result of the NaYF_4_ phase transition from cubic to hexagonal. The hexagonal-phase NaYF_4_ has a much higher fluorescence efficiency as compared to its cubic phase. The PLQY of these heterostructured nanocrystals under 365 nm excitation were as follows: 150 °C (53%), 200 °C (35%), 250 °C (12%), 300 °C—0 min (23%), and 300 °C—60 min (21%) (Supplementary Table 3). Under the excitation of 365 nm, the peak position of heterostructured nanocrystals was 525 nm. The PLQY increased from 12% to 23% during the heterostructured nanocrystal phase-transition process. The fluorescence quantum efficiency of the conventional CsPbBr_3_ nanocrystals was 65%, and fluorescence quenching occurred after CsPbBr_3_ nanocrystals was kept at 300 °C for 60 min (Supplementary Table 3 and Supplementary Table 4). On the other hand, the PLQY of the perovskite within the heterostructured nanocrystals excited by 365 nm was about 21% after being kept at 300 °C for 60 min far higher than that of the conventional CsPbBr_3_ nanocrystals at 300 °C for 60 min (Supplementary Table 4). The conventional CsPbBr_3_ nanocrystals were kept at 300 °C for 60 min, resulting in very small-sized particles and very weak fluorescence (Supplementary Fig. 4). This is because the conventional CsPbBr_3_ nanocrystals shrank in size, dissolved, and their crystal structure was destroyed at 300 °C after 60 min. The heterojunctured nanocrystals have strong fluorescence at the 525 nm peak under 365 nm excitation, which fully indicated that the cubic-phase and hexagonal-phase UCNP have an obvious protective effect on CsPbBr_3_ nanocrystals at high temperature. The hybrid nanocrystals were reheated reheated to different temperatures of 25 °C, 40 °C, 50 °C, 60 °C, 70 °C, 80 °C, 90 °C, 100 °C, 150 °C, and 200 °C, and compared to pure CsPbBr_3_ perovskite QDs. When the temperature was increased to 200 °C, the intensity of CsPbBr_3_ QDs quickly reduced to 2% while the intensity of the hybrid nanocrystals dropped to 71% only, showing the protection of the CsPbBr_3_ QDs by the UCNP at high temperature (Fig. 3d and Supplementary Fig. 14). Relative fluorescence intensity of the heterostructured CsPbBr_3_–NaYF_4_:Yb,Tm hybrid nanocrystals still maintained around 90% during the heating process under 980 nm excitation (Supplementary Fig. 15). The hybrid nanocrystals maintained the luminescent properties of both perovskite QDs and UCNP and improved the stability of perovskite QDs at high temperatures.

### Exploration of the energy-transfer mechanism

The Förster resonance energy transfer (FRET) and the emission reabsorption (ERA) were the two main energy-transfer processes. The changes in fluorescence lifetime are directly associated with FRET, but not with ERA. Efficient FRET between NaYF_4_:Yb,Tm donors and CsPbBr_3_ acceptors will only take place at short distances. Indeed, the fluorescence average lifetime reduction of the blue emission at 478 nm from 0.66 ms of the NaYF_4_:30%Yb,0.5%Tm nanocrystals to 0.29 ms of the CsPbBr_3_–NaYF_4_:Yb,Tm nanocrystals (Supplementary Fig. 16 and Supplementary Table 5) suggested that the energy transfer from NaYF_4_:Yb,Tm to CsPbBr_3_ in the heterostructure follows a typical FRET process^27,41^. The FRET efficiency can be calculated with the equation Eff = 1 − τ_D–A_/τ_D_, where Eff is the energy transfer efficiency, τ_D–A_ is the effective lifetime of a donor conjugated with an acceptor, and τ_D_ is the effective lifetime of a donor in the absence of an acceptor. The FRET efficiency of the CsPbBr_3_–NaYF_4_:Yb,Tm nanocrystals calculated was 56%, which justifies our design for an efficient energy transport strategy. The fluorescence lifetime of the mixed CsPbBr_3_ and NaYF_4_:Yb,Tm is 0.59 ms, which only reduces 0.07 ms compared with the NaYF_4_:30%Yb,0.5%Tm NPs fluorescence lifetime of 0.66 ms, and the efficiency of FRET is calculated to be 11%, far lower than the energy transfer efficiency of FRET in the heterogeneous junction (Supplementary Figs. 16, 17 and Supplementary Tables 5, 6). Therefore, the energy transfer process of the mixed CsPbBr_3_ and NaYF_4_:Yb,Tm is mainly EAU, which is different from that of FRET in heterostructured CsPbBr_3_-NaYF_4_:Yb,Tm hybrid nanocrystals. In addition, the fluorescence lifetime of the perovskites has increased from 18 ns to 0.37 ms (Supplementary Fig. 18 and Supplementary Table 7).

By changing the number of perovskites, we have changed the ratio between the perovskite and UCNP in the heterostructured nanocrystals, and obtained 0.2-fold and fivefold CsPbBr_3_–NaYF_4_:Yb,Tm nanocrystals. The fluorescence lifetime of the onefold heterostructured nanocrystal is 0.29 ms (Supplementary Fig. 19 and Supplementary Table 8). The fluorescence lifetime of the 0.2-fold heterostructured nanocrystal increased to 0.49 ms, while that of the fivefold heterostructured nanocrystal decreased to 0.25 ms (Supplementary Table 8). As compared to the FRET efficiency of the onefold heterostructured nanocrystal, 56%, the FRET efficiency of the 0.2-fold heterostructured nanocrystal was reduced to 25.8%. On the other hand, the FRET efficiency of the fivefold heterostructured nanocrystal increased to 62.1% (Supplementary Table 9). Therefore, it shows that the decrease in the perovskite/UCNP ratio resulted in increased fluorescence lifetime and decreased FRET efficiency. Compared to the fluorescence spectra of pure UCNP under 980 nm excitation, the absorption of the fluorescence emitted from UCNP increases with the increase of the perovskite amount (Supplementary Fig. 20).

### Regulation of optical properties

The heterojunction nanocrystals synthesized by us could undergo anion exchange with Cl^−^ and I^−^ to control the energy transfer and luminescence performance. Under the 980 nm excitation, the 525 nm fluorescence peak emitted by the perovskites within the heterogeneous crystals was shifted to 578 nm after the addition of I^−^. On the other hand, this same peak was shifted to 478 nm after the addition of Cl^−^ (Supplementary Fig. 21A). During the anion exchange in CsPbBr_3_ nanocrystals with the introduction of I^−^ into the heterogeneous crystal, and the fluorescence lifetime of CsPbBr_3_ nanocrystals in the heterojunction became longer. However, the introduction of Cl^−^ into the heterojunction shortened the fluorescence lifetime of CsPbBr_3_ nanocrystals in the heterogeneous crystal^42^, thus affecting the energy transfer efficiency. Under the 365 nm excitation, the 525 nm fluorescence peak emitted from the heterogeneous crystal was blue shifted to 479 nm and red shifted to 578 nm, respectively (Supplementary Fig. 21B). Therefore, this heterojunction nanocrystal structure was able to regulate the energy-transfer process and optical properties from NaYF_4_:Yb,Tm to CsPbBr_3_ nanocrystals via anion exchange. Compared with pure UCNPs, heterogeneous crystals can not only regulate their optical properties through anion exchange but also change the composition of CsPbX_3_ nanocrystals in heterogeneous crystals. With the ratios of perovskites to UCNPs kept unchanged and only the perovskite composition was changed, we have obtained the heterostructured CsPbBr_2_/Cl_1_-NaYF_4_:Yb,Tm nanocrystals and the heterostructured CsPbBr_2_/I_1_-NaYF_4_:Yb,Tm nanocrystals. The size of the heterostructured CsPbBr_2_/Cl_1_-NaYF_4_:Yb,Tm and CsPbBr_2_/I_1_-NaYF_4_:Yb,Tm nanocrystals were 42.3 nm and 39.6 nm, respectively (Supplementary Fig. 24A, B). The irregular small particles of perovskite in the heterostructured nanocrystals were also observed. According to the fluorescence spectrum under the excitation of 980 nm (Supplementary Fig. 24C, D), the emission peak of the perovskites in the heterostructured CsPbBr_2_/Cl_1_-NaYF_4_:Yb,Tm nanocrystals was shifted left to 477 nm, while the emission peak of the perovskites in the heterostructured CsPbBr_2_/I_1_-NaYF_4_:Yb,Tm nanocrystals was shifted right to 542 nm. Under 365 nm excitation, the fluorescence peaks of the heterostructured CsPbBr_2_/Cl_1_-NaYF_4_:Yb,Tm and CsPbBr_2_/I_1_-NaYF_4_:Yb,Tm nanocrystals were 478 nm and 542 nm (Supplementary Fig. 24E, F), respectively. These peaks matched with the upconversion fluorescence peaks of CsPbX_3_ in the heterostructured nanocrystals which indicated that the NaYF_4_:Yb,Tm has a protective effect on the structure of perovskite at high temperature. The fluorescence lifetimes of the heterostructured CsPbBr_2_/Cl_1_-NaYF_4_:Yb,Tm and CsPbBr_2_/I_1_-NaYF_4_:Yb,Tm nanocrystals at the 478 nm peak were 0.23 ms and 0.48 ms, respectively (Supplementary Fig. 24G-H and Supplementary Table 10). Therefore, we have shown that our synthesis strategy is compatible to synthesize heterogeneous junctions composed of different types of UCNPs and perovskites. If the aforementioned three conditions were met, different types of heterogeneous junction products such as PbS-UCNPs, CdS-UCNPs can be obtained.

### Phase transition

**Fig. 4:**
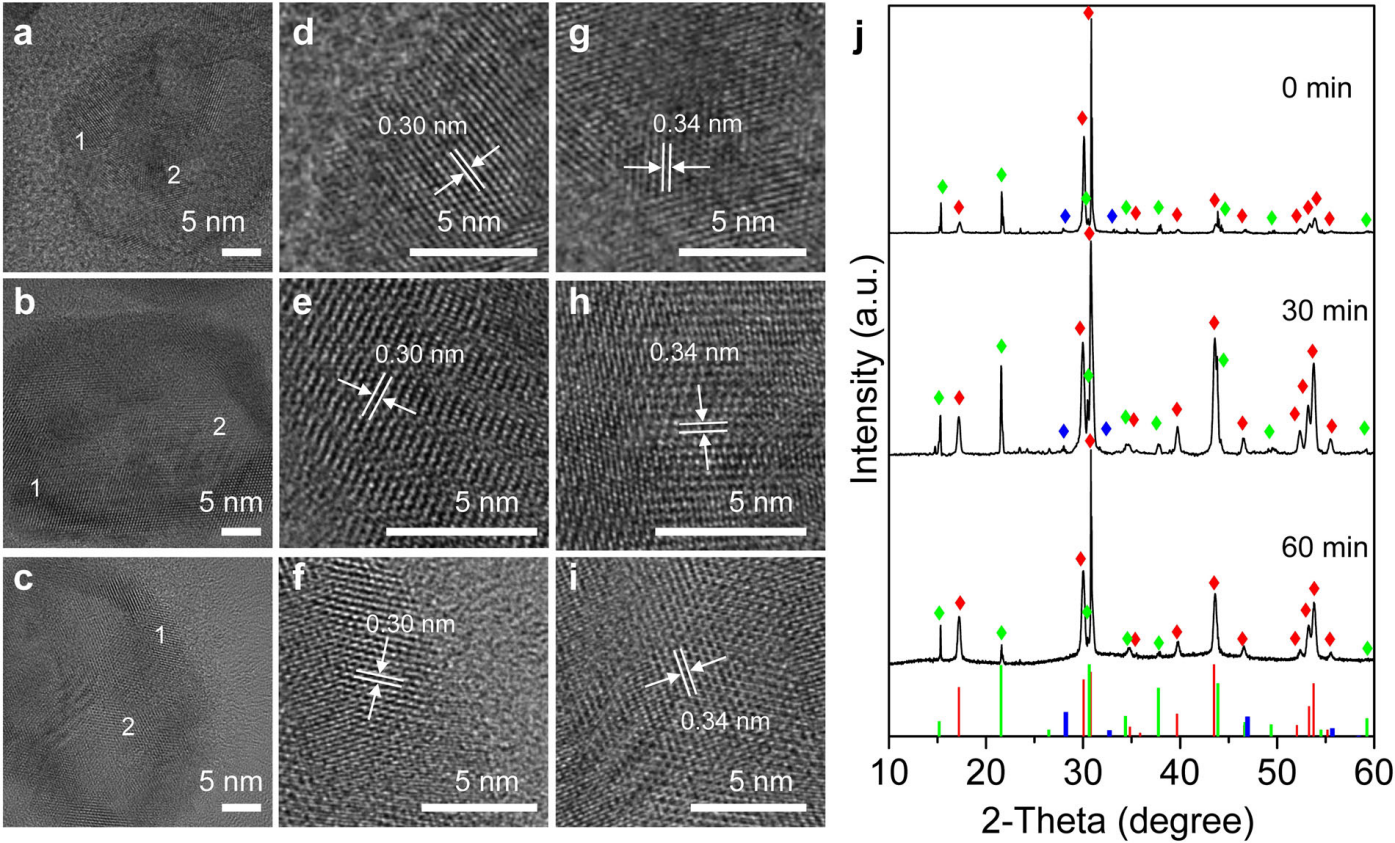
Structural analysis of CsPbBr_3_-NaYF_4_:Yb,Tm hybrid nanocrystals. HRTEM images of heterostructured CsPbBr_3_-NaYF_4_:Yb,Tm nanocrystals collected during the synthesis after heated at 300 °C for different time: **a** 0 min, **b** 30 min, **c** 60 min. **d**–**f** Enlarged images of region 1 in **a**, **b**, and **c**, respectively. **g**–**i** Enlarged images of region 2 in **a**, **b**, and **c**, respectively. **j** X-ray diffraction (XRD) patterns of the three samples. Hexagonal NaYF_4_, red color (JCPDF#00-016-0334), cubic NaYF_4_, blue color (JCPDF #01-077-2042), cubic CsPbBr_3_, green color (JCPDF #00-054-0752).

At 250 °C, the heterojunction nanocrystals mainly contained cubic-phase CsPbBr_3_ crystals and cubic-phase NaYF_4_:Yb,Tm crystals according to the standard data of JCPDS# 00-054-0752 and JCPDS# 00-016-0334 (Supplementary Fig. 25). The 2*θ* of the {100} crystal plane of the cubic-phase CsPbBr_3_ crystals has increased from 15.18° to 15.3°, and the lattice spacing has decreased. On the other hand, the 2*θ* of the {100} crystal plane of the cubic-phase NaYF_4_:Yb,Tm crystals has decreased from 28.2° to 27.8°, which further indicated that the lattice strain was generated by the heterojunction nanocrystals at 250 °C. In order to understand the phase transition during the process of maintaining the temperature at 300 °C for 0 to 60 min, lattice spacing and XRD measurements for the above samples were performed. In this process, as seen in Fig. 4a–i, the lattice distance on the surface of the hybrid nanocrystals was 0.3 nm in accordance with the {110} lattice spacing of hexagonal-phase NaYF_4_ UCNP (JCPDS: 00-016-0334), while the lattice distance in the middle was 0.34 nm corresponding to the {111} lattice spacing of cubic-phase CsPbBr_3_ QDs (JCPDS: 00-054-0752). The high-resolution TEM images confirmed that the two crystals were grown together and well combined which is very important to ensure a highly efficient energy transfer between them when excited at 980 nm. At 300 °C, XRD results in Fig. 4j showed that the nanocrystals mainly contained cubic-phase CsPbBr_3_ crystals and hexagonal-phase NaYF_4_ crystals, according to the standard data of JCPDS# 00-054-0752 and JCPDS# 00-016-0334. Very weak peaks of cubic-phase NaYF_4_ crystals (JCPDS: 01-077-2042) were also observed. With the extension of the thermal insulation time, the cubic-phase NaYF_4_ crystals transformed to hexagonal-phase NaYF_4_ crystals and finally disappeared, resulting in an increase in the relative proportion of hexagonal NaYF_4_ crystals (Supplementary Fig. 26), so only cubic-phase CsPbBr_3_ crystals and hexagonal-phase NaYF_4_ crystals co-existed in the hybrid nanocrystals. The process of phase transition led to the formation of crystal boundary in the nanocrystal structure, which was confirmed with HRTEM results. When cubic-phase NaYF_4_ crystals transformed to the hexagonal phase with high fluorescence efficiency, upconversion fluorescence was enhanced, which was also consistent with the results of fluorescence measurement.

### Stability study

**Fig. 5:**
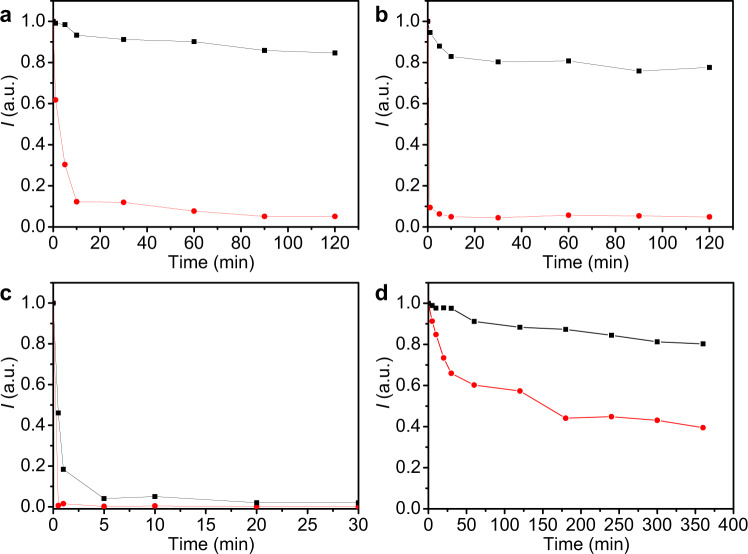
Stability analysis of CsPbBr_3_–NaYF_4_:Yb,Tm hybrid nanocrystals. PL intensity test was used to monitor the stabilities of CsPbBr_3_ (red) and the heterostructure composite of CsPbBr_3_–NaYF_4_:Yb,Tm nanocrystals (black) in cyclohexane and ethanol (v/v = 9: 1) mixed solvent (**a**), cyclohexane and ethanol (v/v = 1: 1) mixed solvent (**b**), water solvent (**c**), and continuous ultraviolet light irradiation (**d**) under 365 nm excitation.

The stability in a mixture solution of cyclohexane and ethanol with a ratio of 9:1 or 1:1 has significantly improved as compared to the conventional CsPbBr_3_ nanocrystals. We have studied the stability of heterostructure composite of CsPbBr_3_-NaYF_4_:Yb,Tm nanocrystals in a mixture solution of cyclohexane and ethanol with a ratio of 9:1 (Fig. 5a and Supplementary Fig. 27) also. The relative fluorescence intensity of the CsPbBr_3_ nanocrystals decreased to 12% at 10 min and 5% at 120 min in the mixture solution. On the other hand, the heterostructure composite of CsPbBr_3_-NaYF_4_:Yb,Tm nanocrystals has managed to maintain a relative fluorescence intensity of 93% at 10 min and 85% at 120 min. This result suggests good stability in a mixture of cyclohexane and ethanol with a ratio of 9:1. Furthermore, the relative fluorescence intensity of the CsPbBr_3_ nanocrystals quickly decreased to 10% at 1 min and 5% at 120 min in a mixture of cyclohexane and ethanol with a ratio of 1:1 (Fig. 5b and Supplementary Fig. 27), whereas the heterostructure composite of CsPbBr_3_-NaYF_4_:Yb,Tm nanocrystals has maintained a relative fluorescence intensity of 95% at 1 min and slowly decrease to 80% after 2 h. This result suggests excellent fluorescence stability in a mixture of cyclohexane and ethanol with a ratio of 1:1. The stability of the heterostructure composite of CsPbBr_3_–NaYF_4_:Yb,Tm nanocrystals in water was still less than ideal. As shown in Fig. 5c, the relative fluorescence intensity of CsPbBr_3_ nanocrystals in water solution has decreased to <1% after 30 s, while the relative fluorescence intensity of the heterostructure composite of CsPbBr_3_-NaYF_4_:Yb,Tm nanocrystals was able to remain at 46% in water solution (Supplementary Fig. 28). Even though the relative fluorescence intensity decreased to about 4% in 5 min eventually, the stability of these heterostructured nanocrystals in the water has shown slight improvement as compared to the conventional CsPbBr_3_ nanocrystals. From the results of solvent stability experiments, it can be inferred that there were bare perovskite crystals on the heterogeneous crystal surface that were not completely covered by NaYF_4_:Yb,Tm nanocrystals. Upon contact with the water molecules, the water molecules infiltrated into the heterogeneous crystal structure along with the perovskite found on the crystal surface. Thus, the perovskite crystal structure in the heterogeneous junction was destroyed and reduced the fluorescence significantly. In addition, we have studied light stability. Under continuous ultraviolet light, the CsPbBr_3_ nanocrystals only have a 40% relative fluorescence intensity after 360 min, while heterostructure composite of CsPbBr_3_–NaYF_4_:Yb,Tm nanocrystals maintained an 80% fluorescence intensity (Fig. 5d and Supplementary Fig. 29). This result suggests that the heterostructured nanocrystals have good light stability.

## Discussion

In summary, heterostructured CsPbBr_3_–NaYF_4_:Yb,Tm nanocrystals were synthesized in one-pot with cubic-phase CsPbBr_3_ QDs embedded in hexagonal-phase NaYF_4_:Yb,Tm UCNP to form a structure of watermelon with multiple seeds, using cubic-phase NaYF_4_:Yb,Tm UCNP as an intermediate transition phase. Different phase crystals can not grow on each other epitaxially to form a heterostructure due to unmatched crystal structure and lattice. Synthesizing heterostructured nanocrystals with two or more different crystal phases remains a challenge. We have successfully demonstrated that cubic-phase perovskite QDs and hexagonal-phase UCNP could be combined into a single nanocrystal by introducing an intermediate transition phase into the synthesis. Cubic-phase UCNP was grown on cubic-phase perovskite QDs to form single nanocrystals, followed by a phase transition from cubic-phase UCNP to hexagonal-phase UCNP by heating to a higher temperature, to obtain monodispersed and heterostructured perovskite–UCNP nanocrystals with multiple cubic-phase perovskite QDs embedded in hexagonal-phase UCNP. Going beyond this, a generic strategy has been developed to synthesize heterostructured nanocrystals with different phases based on phase transition and this could be readily applied to many other materials.

## Methods

### Chemicals and materials

Cesium carbonate (Cs_2_CO_3_, 99.9%), lead bromide (PbBr_2_, 99.9%), lead chloride (PbCl_2_, 99.9%), lead iodide (PbI_2_, 99.9%), yttrium acetate ((CH_3_CO_2_)_3_Y·xH_2_O, 99.9%), ytterbium acetate ((CH_3_CO_2_)_3_Yb·4H_2_O, 99.9%), thulium(III) acetate ((CH_3_CO_2_)_3_Tm·xH_2_O, 99.9%), sodium hydroxide (NaOH, 99%), ammonium fluoride (NH_4_F, 98%), octadecene (ODE, 95%), oleic acid (OA, 90%), oleylamine (OAm, 98%), oleylamine chloride (OAmCl, 99%), oleylamine iodide (OAmI, 99%), cyclohexane (99.9%), and toluene (99.9%) were purchased from Sigma-Aldrich and used as received without further purification.

### Synthesis of CsPbBr_3_ perovskite nanocrystals

Cs-oleate was synthesized by reacting Cs_2_CO_3_ (0.2 g) with OA (0.6 mL) in octadecene (7.5 mL) and pre-heated to 120 °C before injection. PbBr_2_ (0.188 mmol) and 5 mL of ODE were loaded into a 100 mL 3-neck flask, dried under vacuum at 120 °C for 1 h, and mixed with vacuum-dried OAm (0.5 mL) and OA (0.5 mL) under a N_2_ atmosphere. The temperature was raised to 150 °C, and 0.6 mL of Cs-oleate solution was rapidly injected. After 5 s, the reaction mixture was cooled in an ice-water bath. Nanocrystals were precipitated by centrifugation at 12,000 rpm and re-dispersed in 10 mL of cyclohexane. Other samples (CsPbBr_2_Cl_1_ and CsPbBr_2_I_1_) with different ratios of Br/Cl or I = 2/1 were synthesized by the same strategy. The other conditions for synthesis and purification remain the same.

### Synthesis of heterostructured CsPbBr_3_- NaYF_4_: Yb,Tm nanocrystals

First, 0.695 mmol Y(CH_3_CO_2_)_3_, 0.30 mmol Yb(CH_3_CO_2_)_3_, and 0.005 mmol Tm(CH_3_CO_2_)_3_ were mixed with 6 mL of oleic acid and 15 mL of octadecene in a 50-mL flask and heated to 150 °C. After cooled down to room temperature, 10 mL of methanol solution (100 mg NaOH and 150 mg NH_4_F) were slowly added into the flask, and the solution was stirred for 10 min. The solution was subsequently heated to remove methanol and degassed at 120 °C for 20 min. In all, 1 mL of CsPbBr_3_ solution was hot-injected into the precursor solution and then heated to 300 °C for 1 h under N_2_ environment. The product was washed twice with cyclohexane by centrifugation for 10 min and dispersed in 20 mL of cyclohexane for further use. The ratio between CsPbBr_3_ and NaYF_4_: Yb,Tm nanocrystals could be adjusted.

### Synthesis of heterostructured CsPbBr_2_Cl_1_- NaYF_4_: Yb,Tm and heterostructured CsPbBr_2_I_1_- NaYF_4_:Yb,Tm nanocrystals

In total, 0.695 mmol (CH_3_CO_2_)_3_Y, 0.30 mmol (CH_3_CO_2_)_3_Yb, and 0.005 mmol (CH_3_CO_2_)_3_Tm were mixed with 6 mL of oleic acid and 15 mL of octadecene in a 50-mL flask and heated to 150 °C. After cooled down to room temperature, 10 mL of methanol solution (100 mg NaOH and 150 mg NH_4_F) was slowly added into the flask, and the solution was stirred for 10 min. The solution was subsequently heated to remove methanol and degassed at 120 °C for 20 min. In all, 1 mL of CsPbBr_2_I_1_ or CsPbBr_2_I_1_ solution was hot-injected into the precursor solution and then heated to 300 °C for 1 h under N_2_ environment. The product was washed twice with cyclohexane by centrifugation for 10 min and dispersed in 20 mL of cyclohexane for further use.

### Anion exchange of heterostructured CsPbBr_3_- NaYF_4_: Yb,Tm nanocrystals

The anion-exchange reactions were performed in a 10-mL glass bottle. The heterostructured CsPbBr_3_- NaYF_4_: Yb,Tm nanocrystals (5 mL, 0.05 mol/L) were used as the precursor. For anion exchange, OAmI or OAmCl (20 mg) was dissolved in cyclohexane (20 mL) as the anion source, and add 850 μL of OAmI or 700 μL of OAmCl solution into the heterostructured nanocrystals solution (5 mL) to achieve anion exchange. Anion exchange could be completed in a time period ranging from tens of seconds to a few minutes. After the reaction, the emission peaks of the solution were detected by a spectrofluorometer.

### Characterization

TEM was performed on an FEI Tecnai G2 F20 electron microscope operating at 200 kV. Low-voltage high-resolution STEM measurement was carried out on a double-aberration-corrected Titan^TM^ cubed G2 60−300 S/TEM equipped with Super-XTM technology. The available point resolution is ~0.1 nm at an operating voltage of 60 kV. XRD was measured with a Bruker AXS D8 X-ray diffractometer equipped with monochromatized Cu Kα radiation (*λ* = 1.5418 Å). XPS was performed using an achromatic Al Kα source (1486.6 eV) and a double-pass cylindrical mirror analyzer (ULVACPHI 5000 Versa Probe) (Japan). Heterostructured nanocrystals were dissolved in cyclohexane solution at a concentration of 0.05 mol/L, and the upconversion fluorescence spectrum, down-conversion fluorescence spectrum, absorption spectrum, excitation spectrum, and fluorescence lifetime were tested and characterized. Ultraviolet and visible absorption (UV–vis) spectra were recorded with a Shimadzu UV-3600 plus spectrophotometer equipped with an integrating sphere under ambient conditions. Photoluminescence excitation and emission spectra and fluorescence decays were recorded on a FLS980 spectrometer (Edinburgh) equipped with both continuous xenon (450 W) and pulsed flash lamps. Upconversion fluorescence emission spectra were acquired under 980 nm excitation with a CW diode laser (2 W/cm^2^). Measurement of absolute up- and down-conversion PLQY of nanocrystals was performed using a standard barium sulfate-coated integrating sphere (Edinburgh). The sample chamber was mounted to a FLS980 spectrophotometer. Lifetime was measured with a customized UV to mid-infrared steady-state and phosphorescence lifetime spectrometer (FSP920-C, Edinburgh) equipped with a digital oscilloscope (TDS3052B, Tektronix) and a tunable mid-band Optical Parametric Oscillator pulsed laser as the excitation source (410–2400 nm, 10 Hz, pulse width ≤5 ns, Vibrant 355II, OPOTEK). Fluorescence decay was measured on a Nikon Ni-U Microfluorescence Lifetime system with a 375 nm picosecond laser and a time-correlated single-photon counting system at room temperature.

## Supplementary information

Supplementary Information

## Data Availability

The data that support the findings of this study are available from the corresponding author upon reasonable request.
