# Supplementary material for: NIR-excitable heterostructured upconversion perovskite nanodots with improved stability
Source: Nat Commun. 2021 Jan 11;12:219. doi: 10.1038/s41467-020-20551-z (PMC7801668; doi:10.1038/s41467-020-20551-z)
Supplement: Supplementary file 1 — Supplementary Information [file 41467_2020_20551_MOESM1_ESM.pdf]

Supplementary Information

NIR-excitable heterostructured upconversion perovskite nanodots with improved stability

*Longfei Ruan [1], Yong Zhang [1,2]**

[1] Department of Biomedical Engineering, Faculty of Engineering, National University of Singapore, Singapore 117583

[2] NUS Graduate School for Integrative Sciences and Engineering, National University of Singapore, Singapore 117456

* Corresponding author:
Prof. Yong Zhang
Department of Biomedical Engineering
Faculty of Engineering, Block E4 #04-08
National University of Singapore
4 Engineering Drive 3, Singapore 117583
Phone: +65-65164871
Fax: +65-68723069
Email: biezy@nus.edu.sg

Supplementary Note 1. Supplemental data for CsPbBr3 QDs

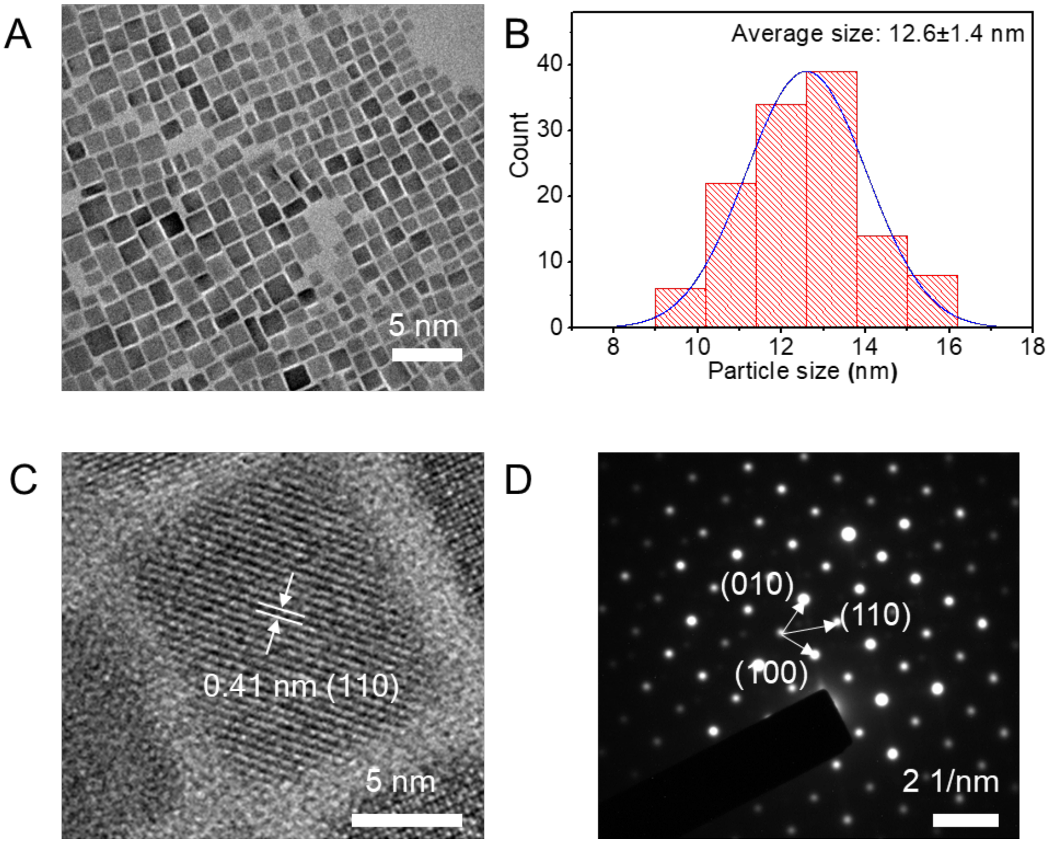

Supplementary Figure 1. (A) TEM image of $CsPbBr_3$ QDs, (B) Histogram representing the statistical distribution of $CsPbBr_3$ QDs in A (average size: 12.6 ± 1.4 nm), (C) HRTEM image of $CsPbBr_3$ QDs, (D) Selected area electron diffraction (SAED) image of $CsPbBr_3$ QDs.

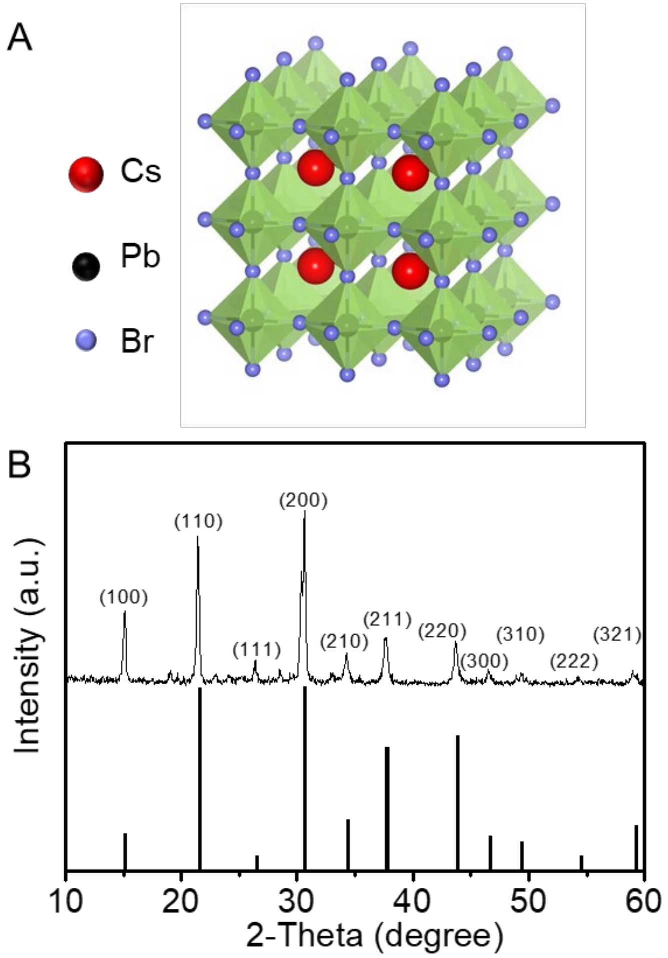

Supplementary Figure 2. (A) The crystal structure and (B) XRD pattern of $CsPbBr_3$ QDs. Standard PXRD of cubic phase $CsPbBr_3$ (JCPDF #00-054-0752).

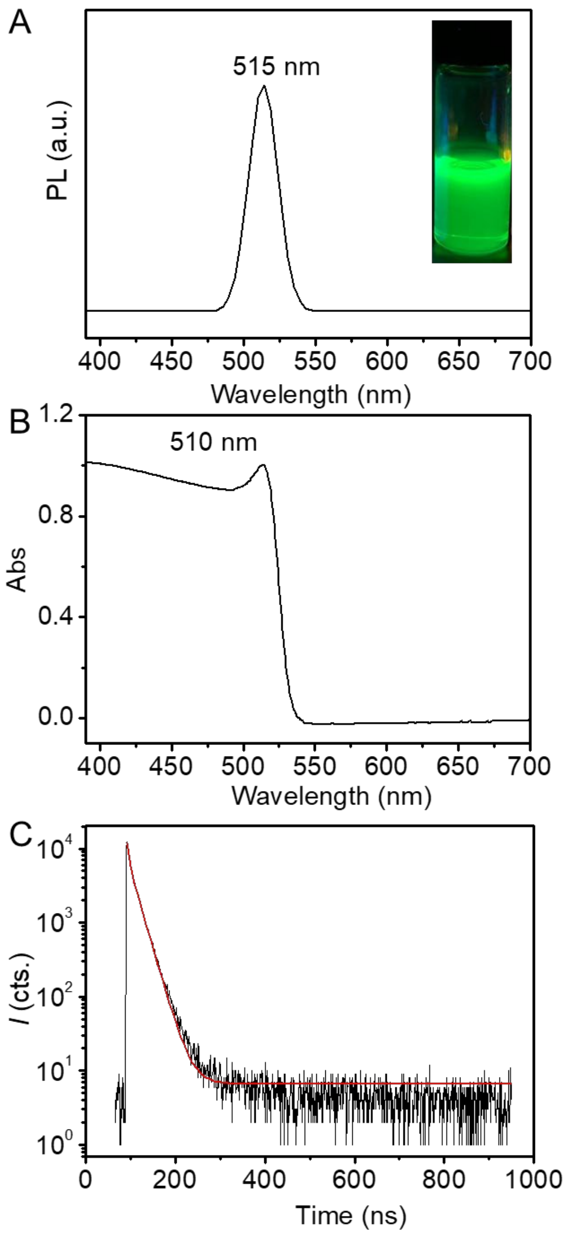

Supplementary Figure 3. (A) Fluorescence emission spectra of $CsPbBr_3$ QDs under 365 nm excitation (the illustration in A shows $CsPbBr_3$ QDs dispersed in cyclohexane under 365 nm excitation at room temperature), (B) UV-Vis absorption spectra of $CsPbBr_3$ QDs, (C) Time-resolved fluorescence decay and fitting curve of $CsPbBr_3$ QDs (Emission at 515 nm) under 365 nm exaction.

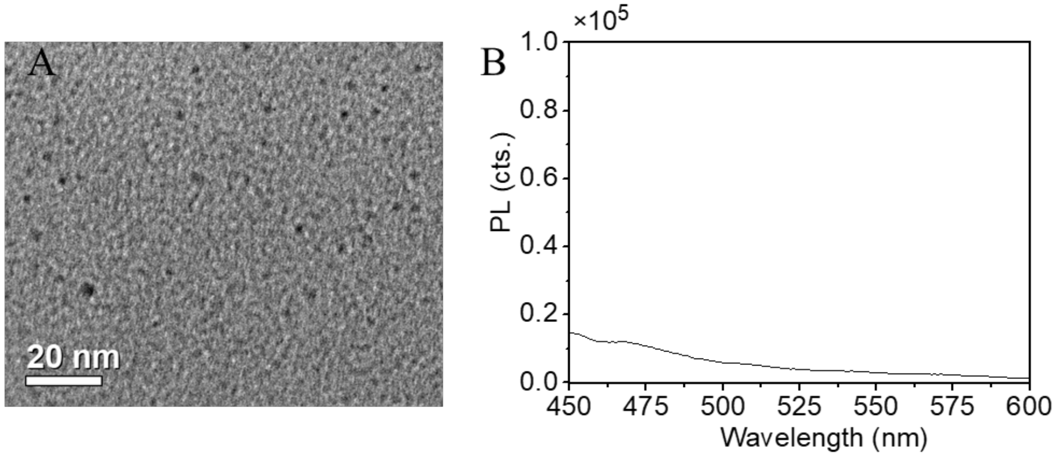

Supplementary Figure 4. (A) TEM image and (B) Fluorescence spectra under 365 nm excitation of $CsPbBr_3$ nanocrystals maintained at a temperature of 300 °C for 1 hour.

Supplementary Note 2. Supplemental data for $NaYF_4$:30%Yb,5%Tm UCNP

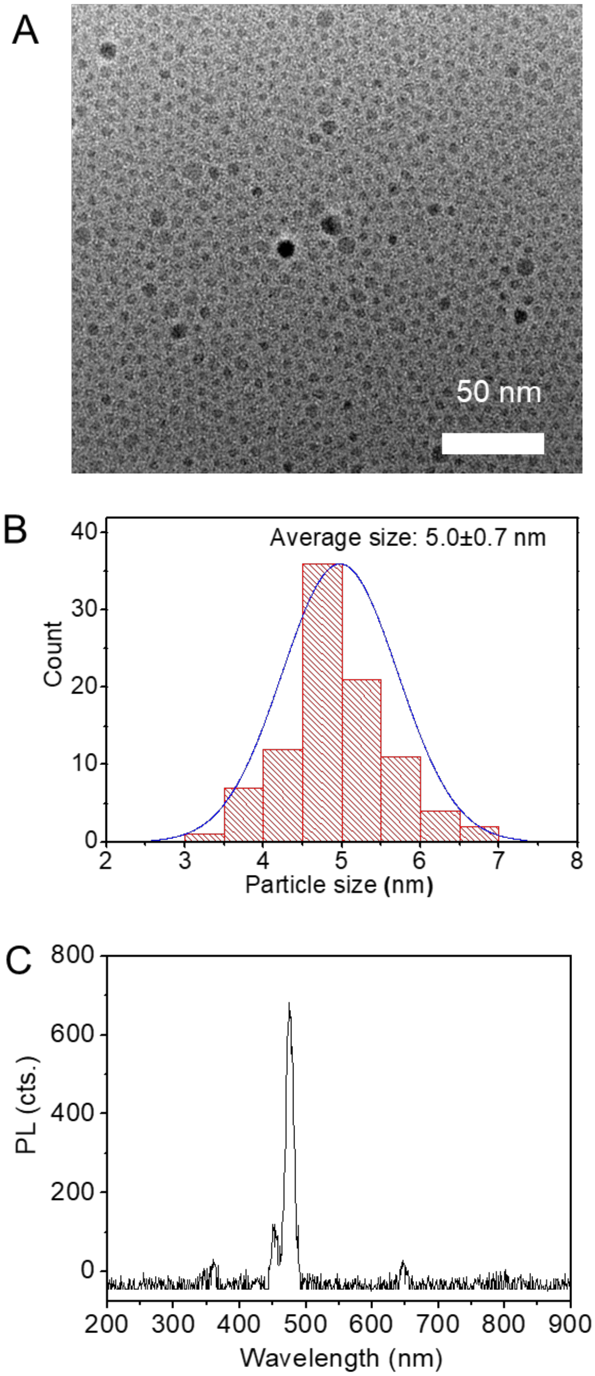

Supplementary Figure 5. (A) TEM image of $NaYF_4$:30%Yb,0.5%Tm nanocrystals at 300 °C for 0 min, (B) Histograms representing statistical distribution of the nanocrystals in A, (C) Fluorescence emission spectra of $NaYF_4$:30%Yb,5%Tm nanocrystals at 300 °C for 0 min under 980 nm excitation.

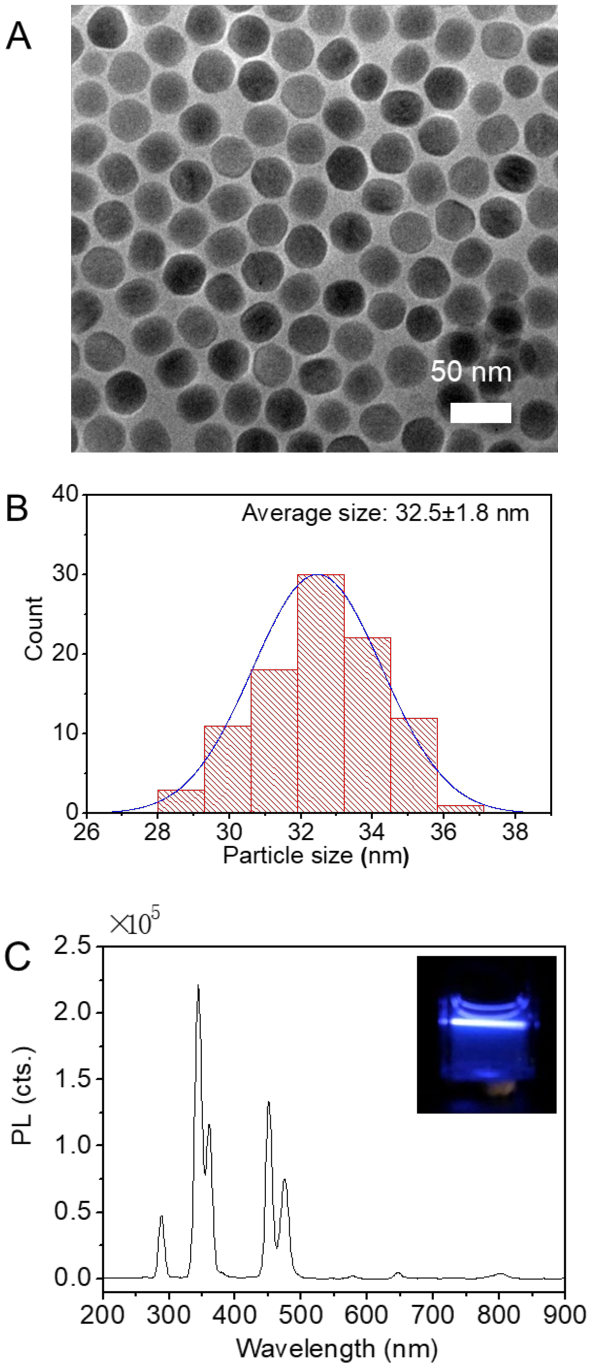

Supplementary Figure 6. (A) TEM image of $NaYF_4$:30%Yb,0.5%Tm nanocrystals at 300 ℃ for 60 min, (B) Histograms representing statistical distribution of the nanocrystals in A, (C) Fluorescence emission spectra of $NaYF_4$:30%Yb,5%Tm nanocrystals at 300 ℃ for 60 min under 980 nm excitation (the illustration in C shows $NaYF_4$:30%Yb,5%Tm nanocrystals dispersed in cyclohexane under 980 nm excitation at room temperature).

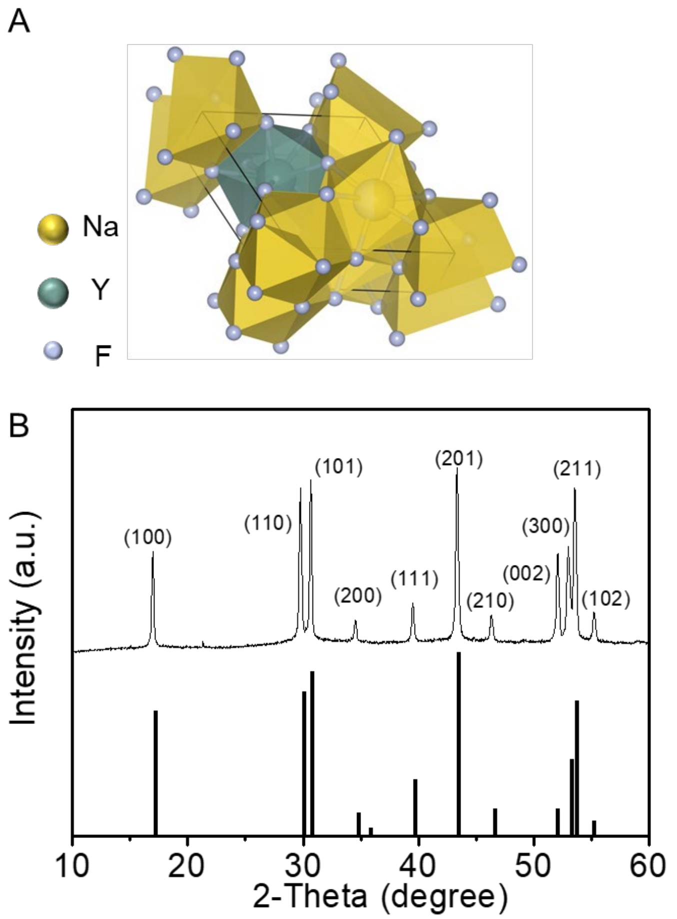

Supplementary Figure 7. (A) Crystal structure of hexagonal $NaYF_4$, (B) XRD pattern of $NaYF_4$:30%Yb,0.5%Tm nanocrystals at 300 °C for 60 min. Standard PXRD of hexagonal $NaYF_4$:Yb/Tm nanocrystals (PDF#01-072-7929).

Supplementary Note 3. Supplemental data for heterostructured $CsPbBr_3$-$NaYF_4$:Yb,Tm hybrid nanocrystals

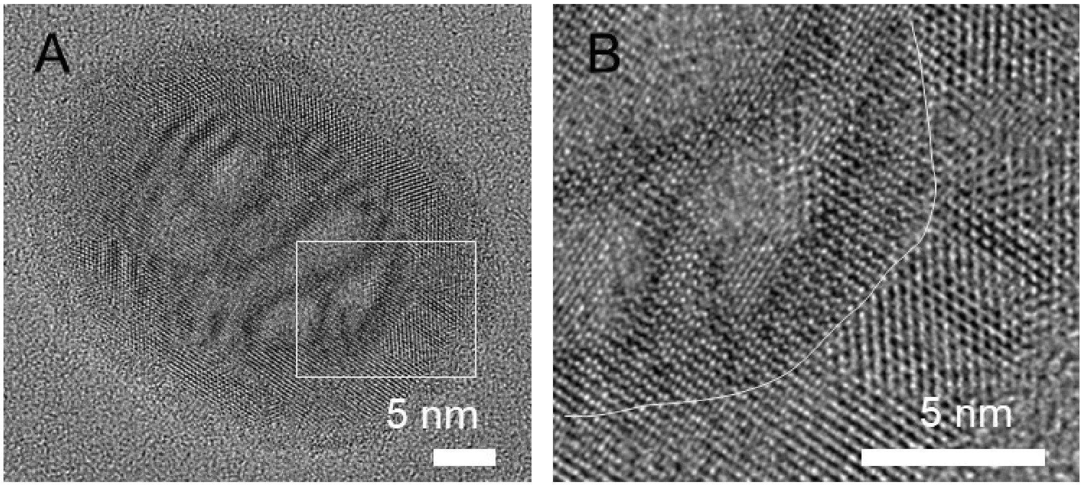

Supplementary Figure 8. (A) HRTEM images of the heterostructured $CsPbBr_3$-$NaYF_4$:Yb,Tm nanocrystals collected during the synthesis after heated at 300 °C for 60 min; (B) A larger view of the square area in Figure 2A.

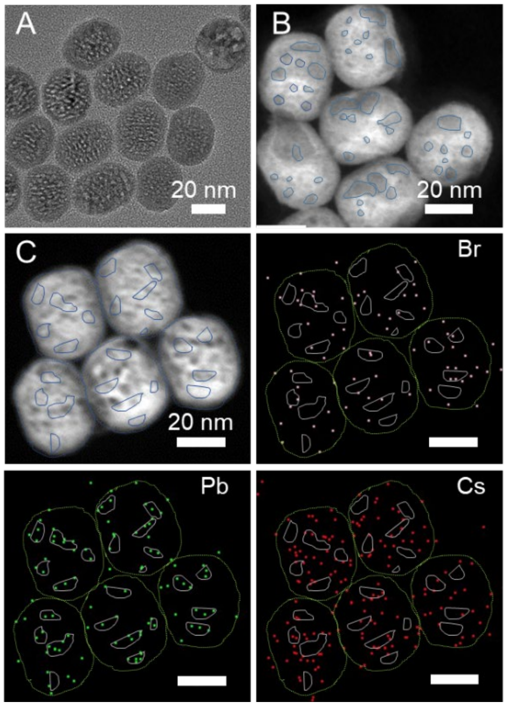

Supplementary Figure 9. (A) TEM and (B) STEM images of the heterostructured $CsPbBr_3$ $NaYF_4$:Yb,Tm nanocrystals; (**C**) Elemental mapping (Cs, Pb and Br) of the heterostructured $CsPbBr_3$-$NaYF_4$:Yb,Tm nanocrystals. Scale bars in C, 20 nm.

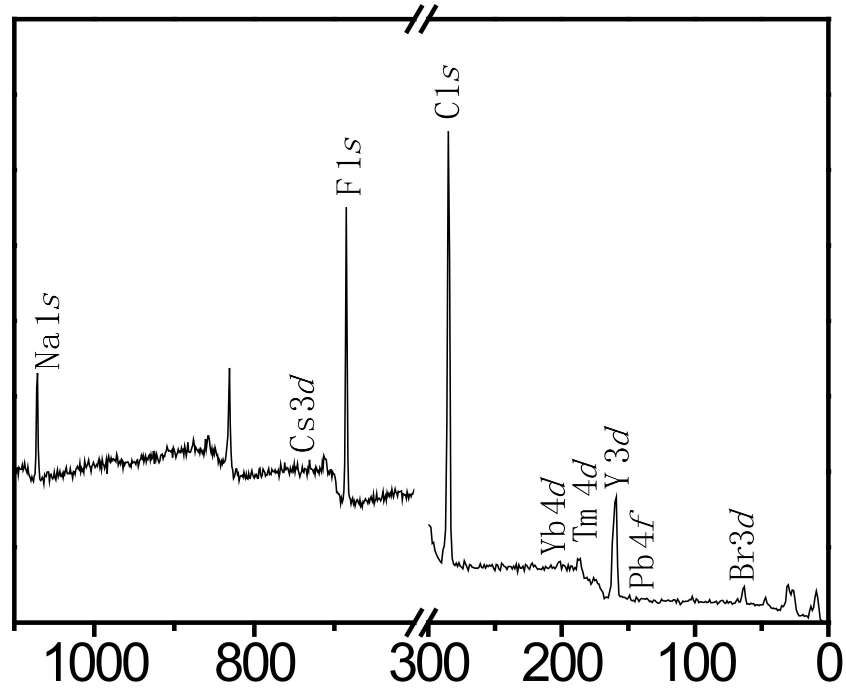

Supplementary Figure 10. X-ray photoelectron spectroscopy (XPS) spectra of heterostructured $CsPbBr_3$-$NaYF_4$:Yb,Tm hybrid nanocrystals. The presence of Cs, Pb Br, F, Na, Y, Tm and Yb in the nanocrystals is confirmed.

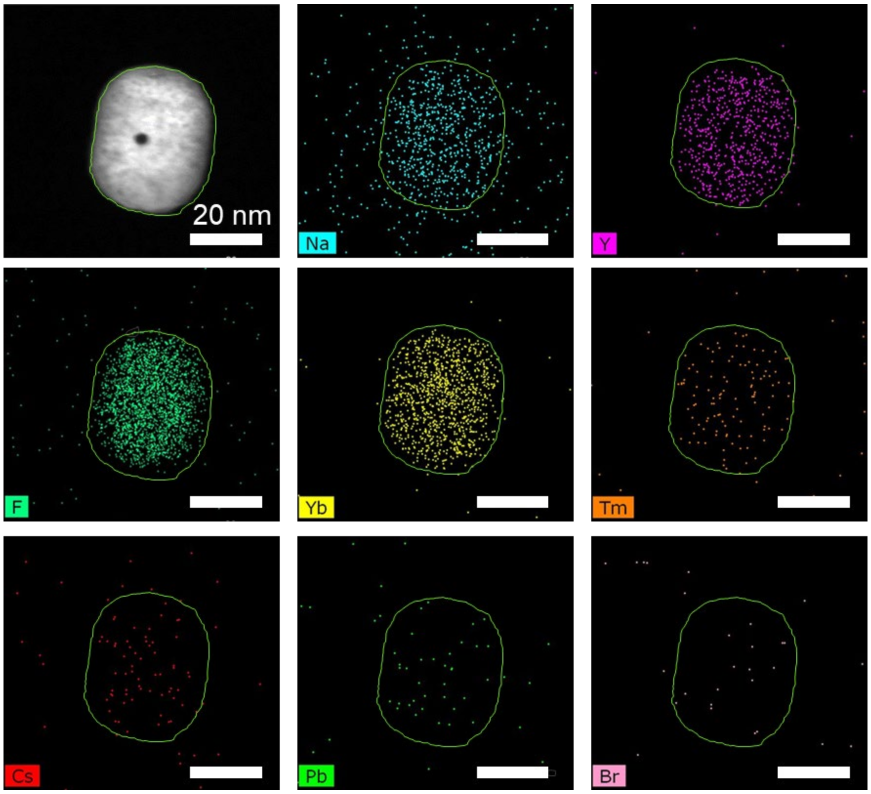

Supplementary Figure 11. Elemental mapping of heterostructured $CsPbBr_3$-$NaYF_4$:Yb,Tm hybrid nanocrystals, all scale bars represent 20 nm.

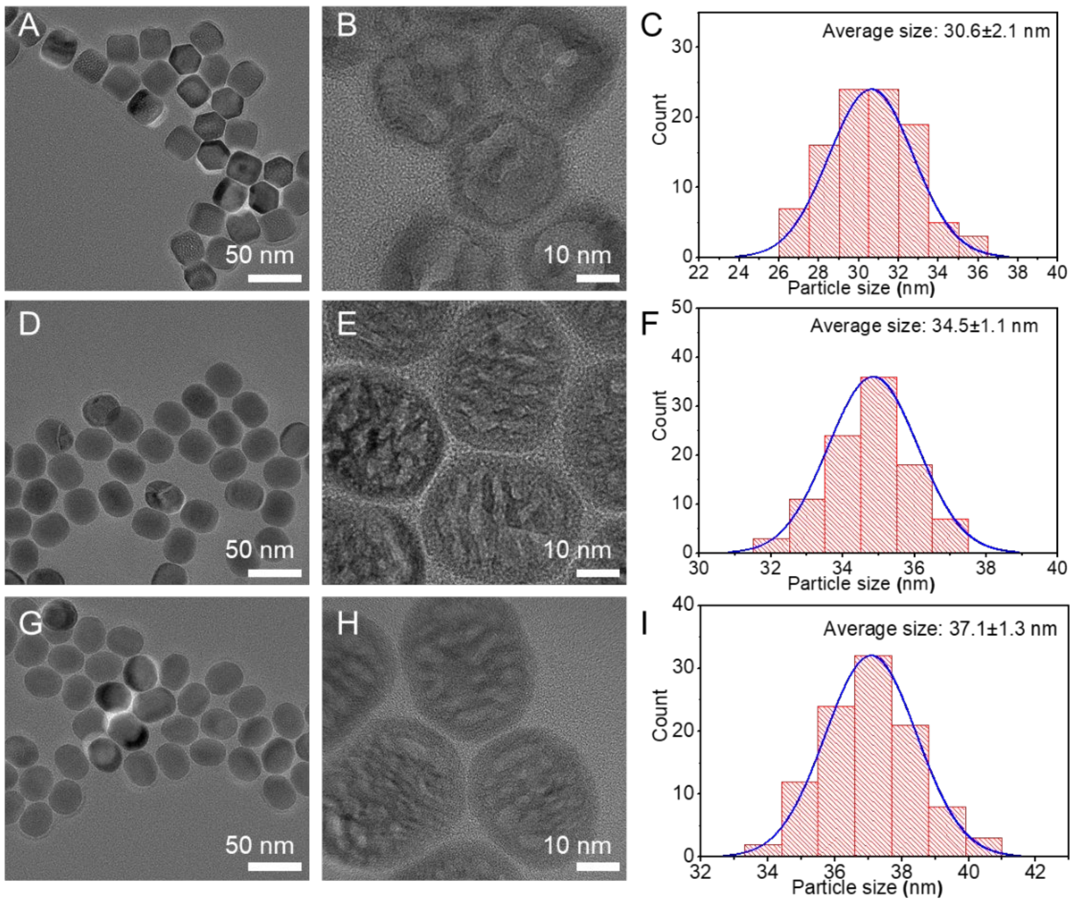

Supplementary Figure 12. TEM and HRTEM images of heterostructured $CsPbBr_3$-$NaYF_4$:Yb,Tm hybrid nanocrystals at 300 °C for different insulation time. (A) and (B) 0 min, (D) and (E) 30 min, (G) and (H) 60 min, (C) (F) and (I) Histograms representing statistical distribution of the nanocrystals in A, D, and G.

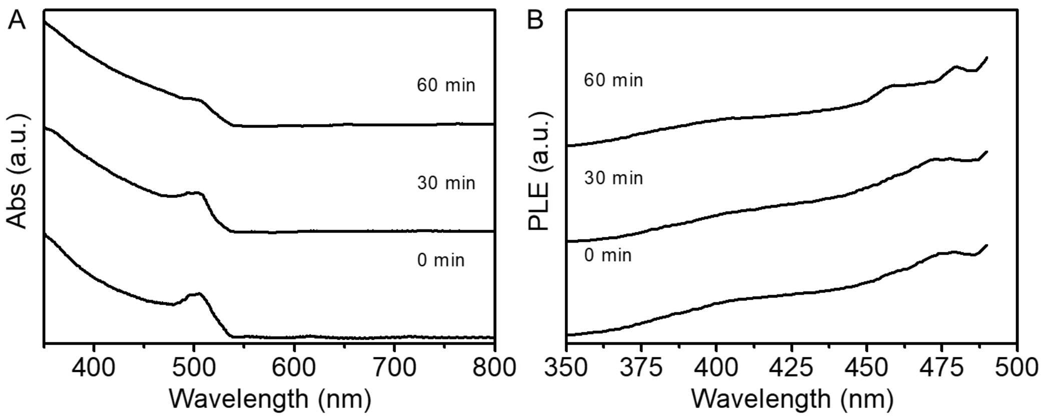

Supplementary Figure 13. UV-Vis absorption spectra (A) and PL excitation spectra (B) of the heterostructure composite of $CsPbBr_3$-$NaYF_4$:Yb,Tm nanocrystals at 300 ℃ at different incubation time.

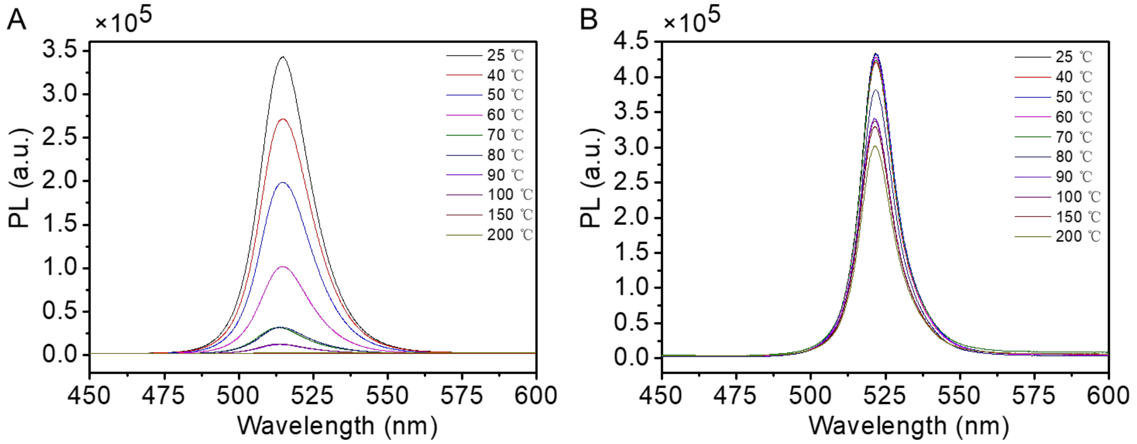

Supplementary Figure 14. (A) and (B) Fluorescence emission spectra of $CsPbBr_3$ QDs and heterostructured $CsPbBr_3$-$NaYF_4$:Yb,Tm hybrid nanocrystals (under UV excitation at 365 nm) when heated at different temperatures.

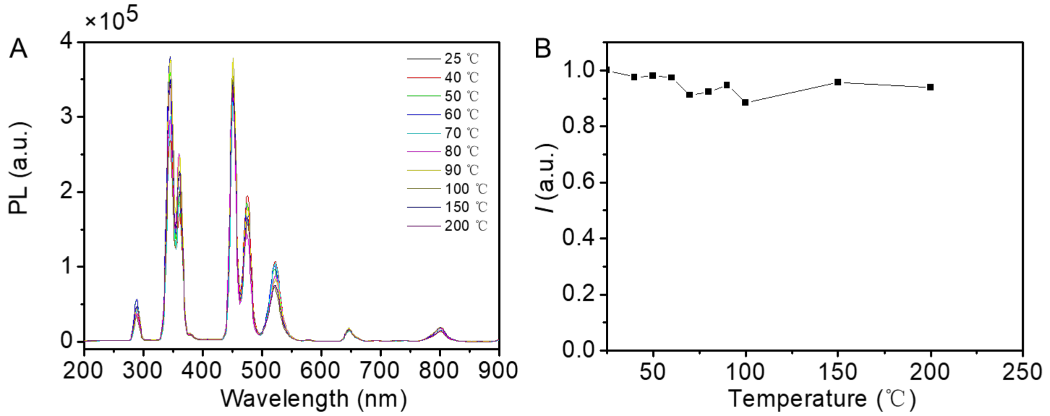

Supplementary Figure 15. (A) and (B) Temperature-dependent PL spectra and relative PL intensities of the heterostructured $CsPbBr_3$-$NaYF_4$:Yb,Tm hybrid nanocrystals under 980 nm excitation.

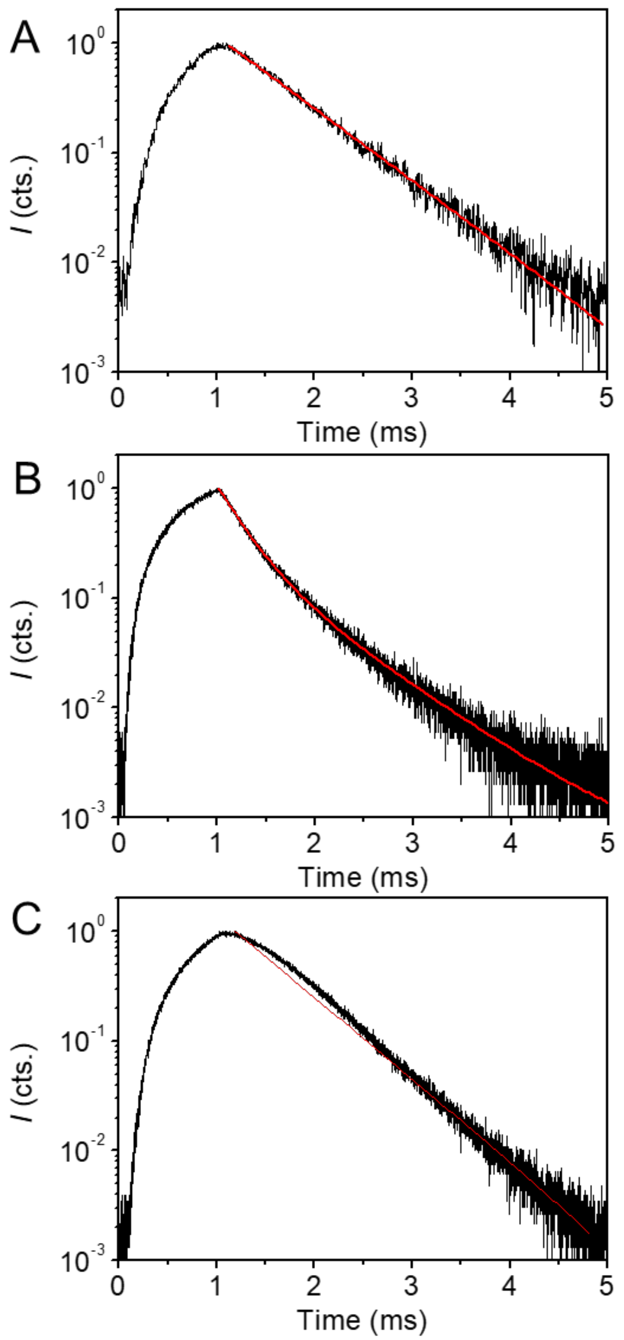

Supplementary Figure 16. Time-resolved fluorescence decay and fitting curve of $NaYF_4$:30%Yb,0.5%Tm nanocrystals (A), heterostructure composite of $CsPbBr_3$-$NaYF_4$:Yb,Tm nanocrystals (B) and $CsPbBr_3$/ $NaYF_4$: 30%Yb,0.5%Tm mixture (C) under 980 nm excitation (monitored at the emission of 478 nm). A curve (red) was fitted for (A), (B), and (C) respectively.

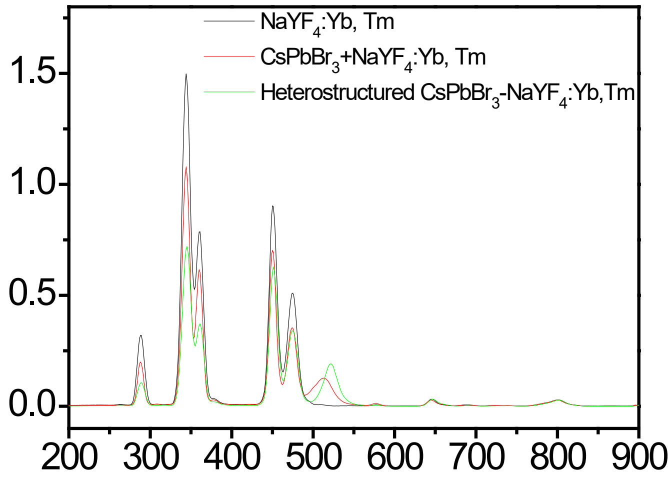

Supplementary Figure 17. Fluorescence spectra of the heterostructured $CsPbBr_3$-$NaYF_4$:Yb,Tm hybrid nanocrystals ($C_{NaYF4:Yb,Tm}$ =0.05 moL/L), $NaYF_4$:30%Yb, 0.5%Tm nanocrystals (0.05 moL/L) at 300°C for 60 mins, and $CsPbBr_3$ QDs mixed with $NaYF_4$: Yb/Tm nanocrystals under 980 nm excitation. The concentration ratio of $CsPbBr_3$ to $NaYF_4$:Yb,Tm nanocrystals was 59:1 in both the heterostructured composites and mixed materials.

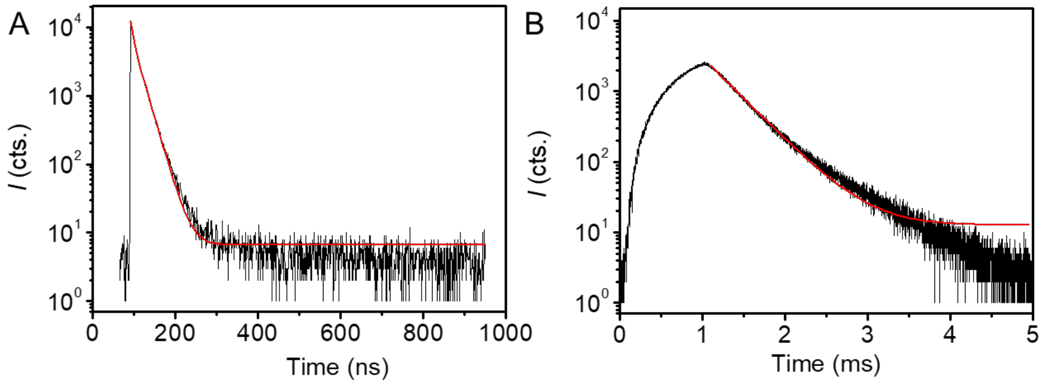

Supplementary Figure 18. Time-resolved fluorescence decay curve of (A) $CsPbBr_3$ QDs under 365 nm excitation (reference taken from the 520 nm emission) and (B) heterostructured $CsPbBr_3$-$NaYF_4$:Yb,Tm nanocrystals under 980 nm excitation (reference taken from the 525 nm emission). A curve (red) was fitted in (A) and (B).

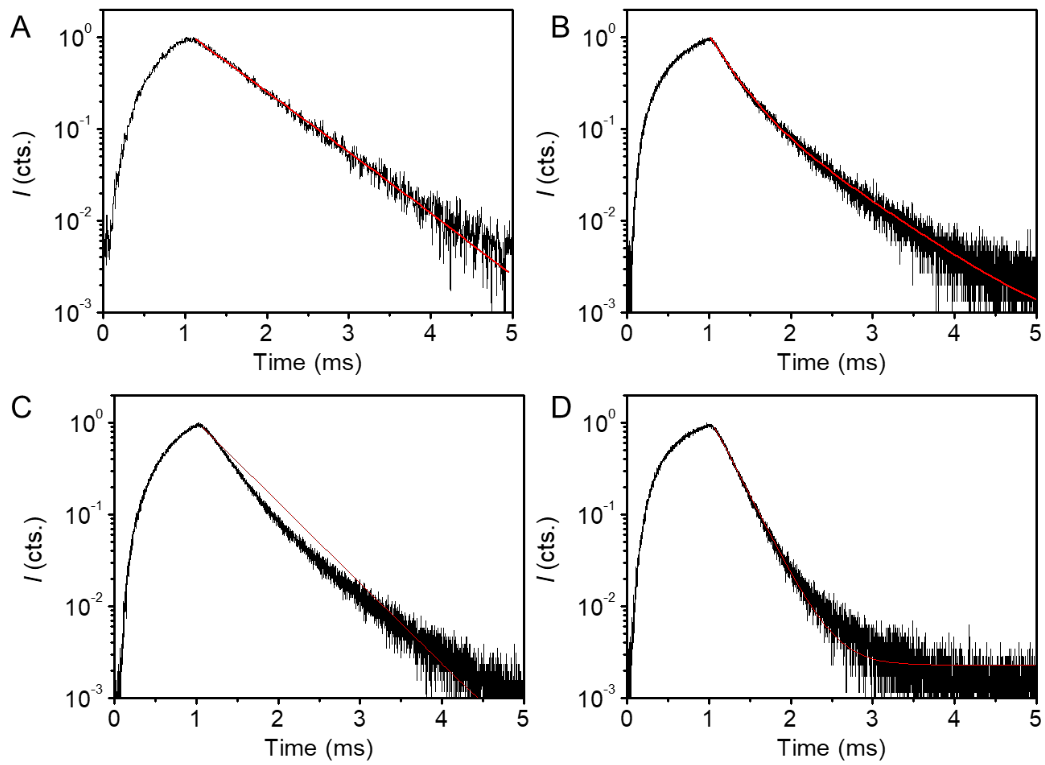

Supplementary Figure 19. Time-resolved fluorescence decay of $NaYF_4$:30%Yb,0.5%Tm nanocrystals (A), heterostructure composite of $CsPbBr_3$-$NaYF_4$:Yb,Tm nanocrystals (B), 0.2 fold heterostructure composite of $CsPbBr_3$-$NaYF_4$:Yb,Tm nanocrystals (C), and 5 fold heterostructure composite of $CsPbBr_3$-$NaYF_4$:Yb,Tm nanocrystals (D) under 980 nm excitation (monitored at the emission of 478 nm). A curve (red) was fitted for (A) , (B), (C), and (D) respectively.

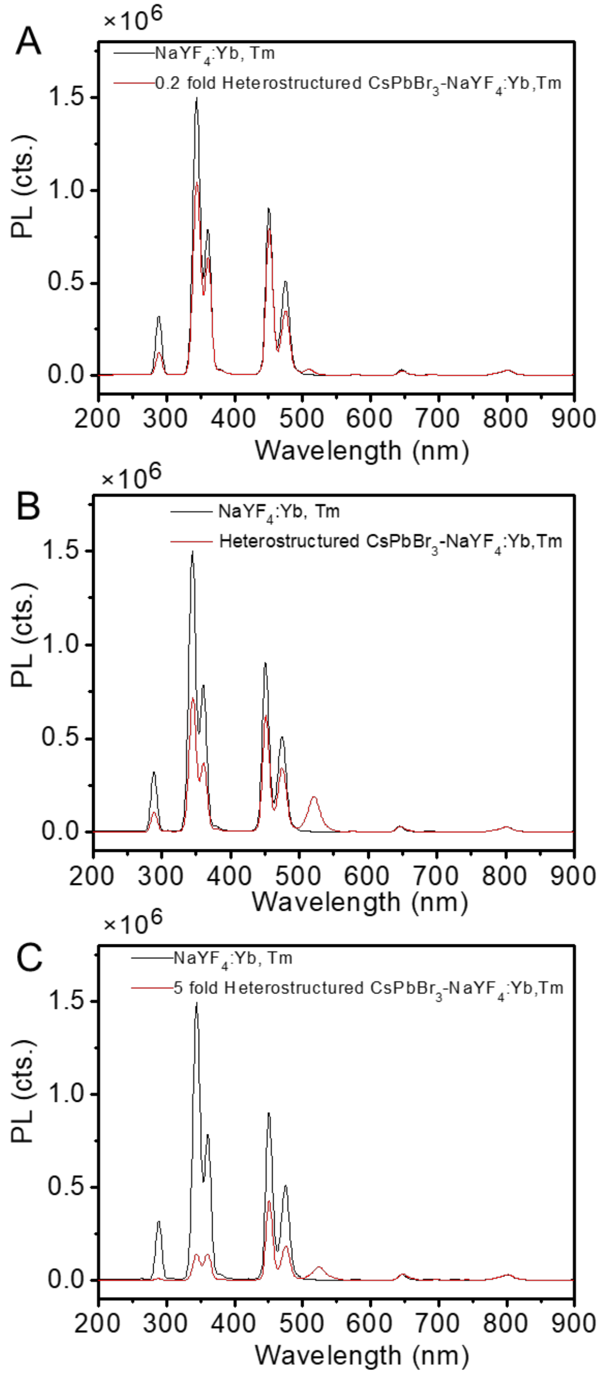

Supplementary Figure 20. Fluorescence spectra of the $NaYF_4$: 30%Yb, 0.5%Tm nanocrystals and the heterostructured $CsPbBr_3$-$NaYF_4$:Yb,Tm hybrid nanocrystals by adding different folds of perovskites in the reaction under 980 nm excitation: (A) 0.2 fold, (B) 1 fold, and (C) 5 fold.

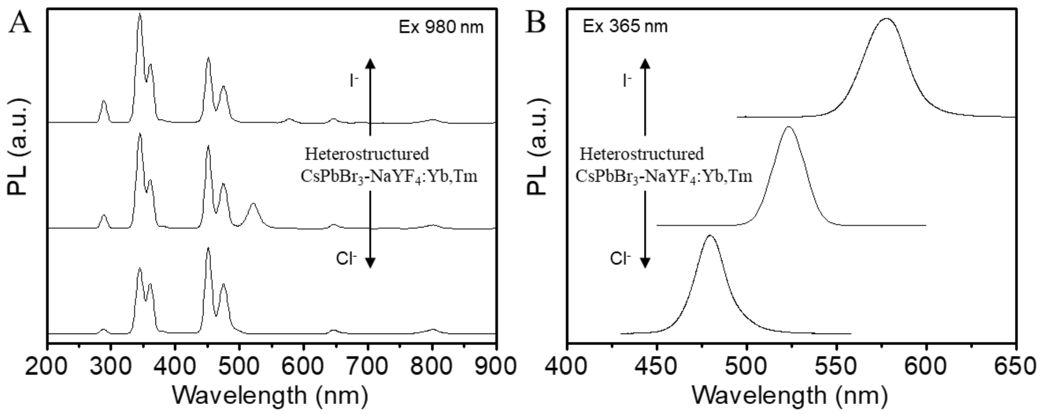

Supplementary Figure 21. Fluorescence spectra under 980 nm (A) and 365 nm (B) excitation of the heterostructure composite of $CsPbBr_3$-$NaYF_4$:Yb,Tm nanocrystals by regulating the halogen ion dopants via anion exchange.

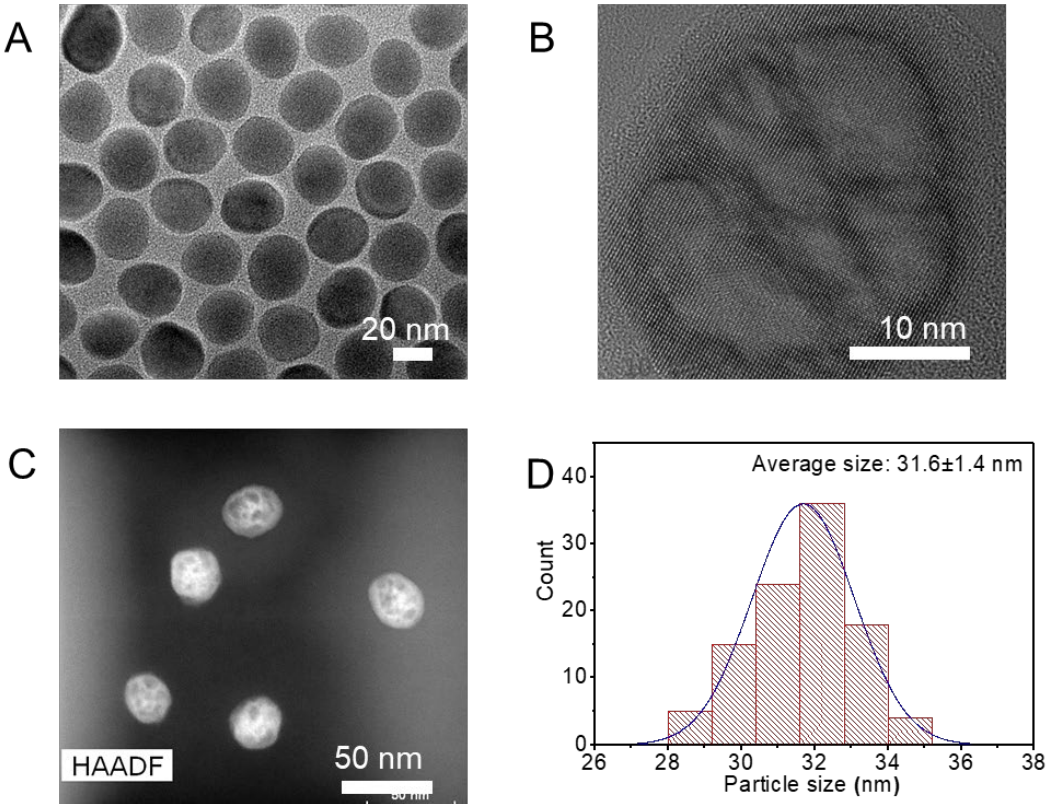

Supplementary Figure 22. (A) TEM, (B) HRTEM and (C) HAADF-STEM images of heterostructured $CsPbBr_3$-$NaYF_4$:Yb,Tm hybrid nanocrystals with 5 times higher concentration of $CsPbBr_3$ QDs, (D) statistical distribution histograms of the nanocrystals in A.

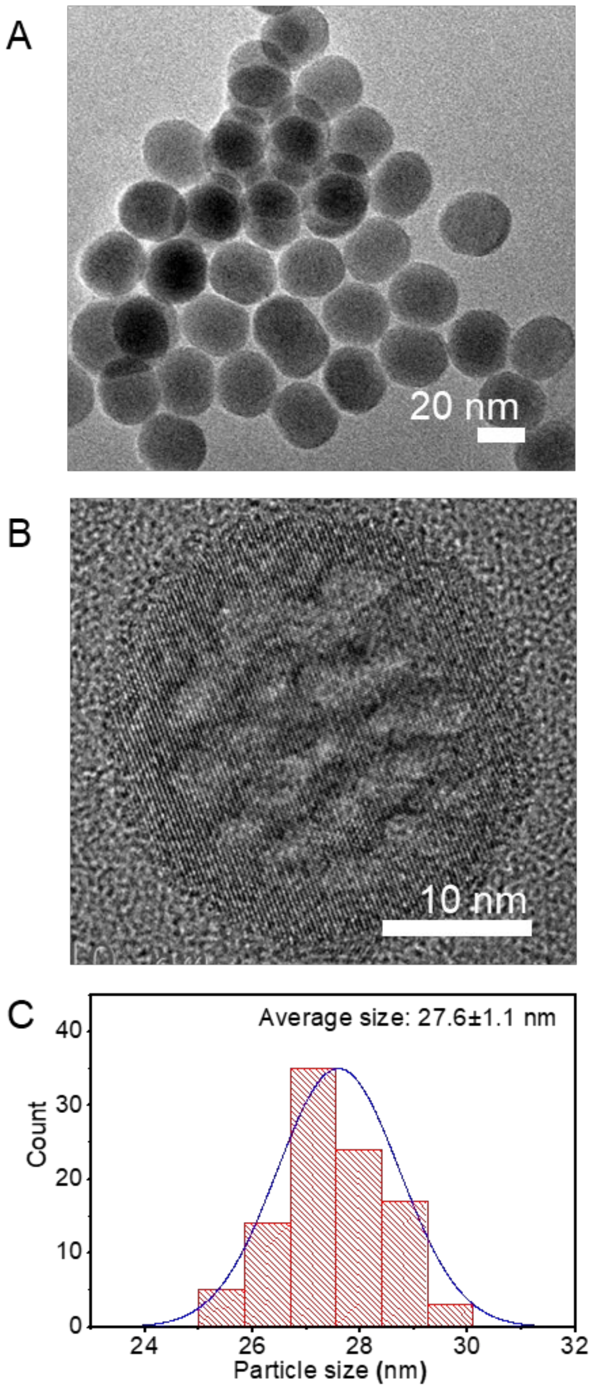

Supplementary Figure 23. (A) TEM and (B) HRTEM images of heterostructured $CsPbBr_3$-$NaYF_4$:Yb,Tm hybrid nanocrystals with 10 times higher concentration of $CsPbBr_3$ QDs, (C) statistical distribution histograms of the nanocrystals in A.

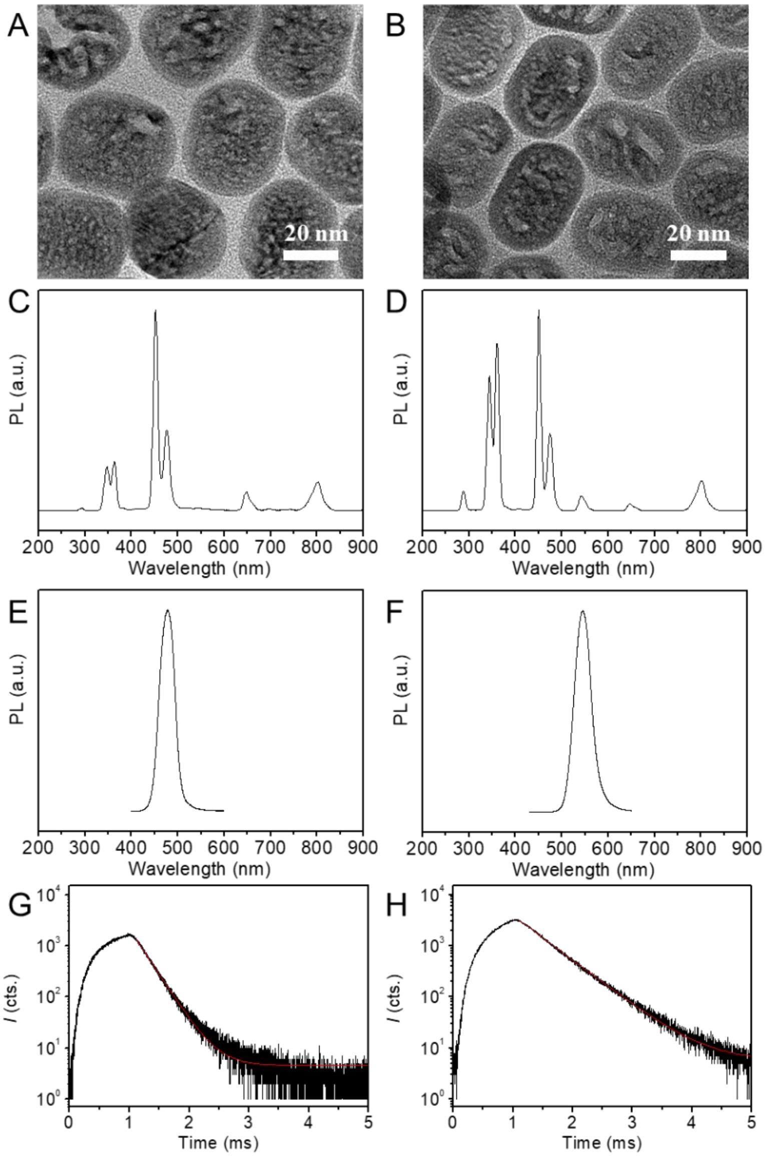

Supplementary Figure 24. TEM image of the heterostructured $CsPbBr_2/Cl_1$-$NaYF_4$:Yb,Tm nanocrystals (A) and the heterostructured $CsPbBr_2/I_1$-$NaYF_4$:Yb,Tm nanocrystals (B); Fluorescence spectra of the heterostructured $CsPbBr_2/Cl_1$-$NaYF_4$:Yb,Tm nanocrystals (C) and the heterostructured $CsPbBr_2/I_1$-$NaYF_4$:Yb,Tm nanocrystals (D) under 980 nm excitation; Fluorescence spectra of the heterostructured $CsPbBr_2/Cl_1$-$NaYF_4$:Yb,Tm nanocrystals (E) and the heterostructured $CsPbBr_2/I_1$-$NaYF_4$:Yb,Tm nanocrystals (F) under 365 nm excitation; Time-resolved fluorescence decay of the heterostructured $CsPbBr_2/Cl_1$-$NaYF_4$:Yb,Tm nanocrystals (G) and the heterostructured $CsPbBr_2/I_1$-$NaYF_4$:Yb,Tm nanocrystals (H) under 980 nm excitation

(reference taken from the 478 nm emission). A curve (red) was fitted for (G) and (H) respectively.

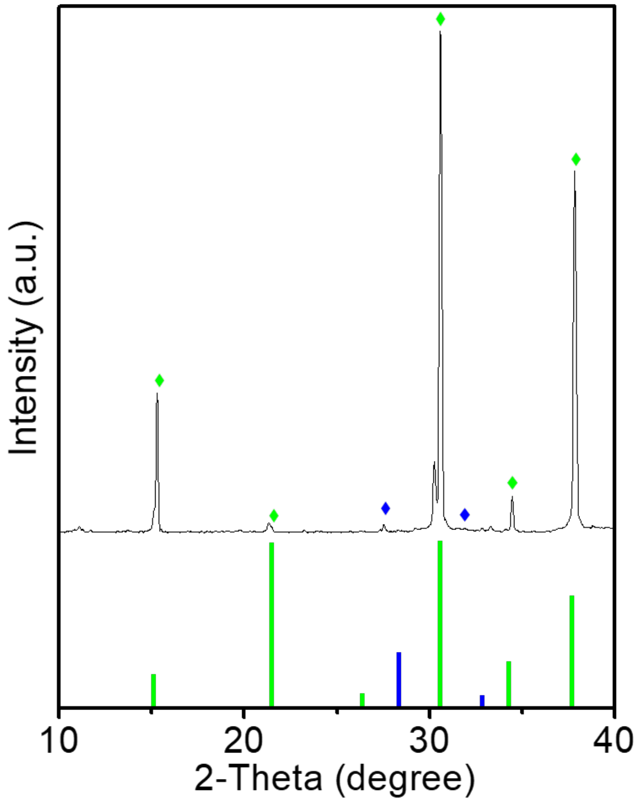

Supplementary Figure 25. X-ray diffraction (XRD) patterns of the heterostructured $CsPbBr_3$-$NaYF_4$:Yb,Tm nanocrystals collected during the synthesis after heated at 250 °C. The cubic $NaYF_4$ is denoted in blue (JCPDF #01-077-2042), while the cubic $CsPbBr_3$ is denoted in green color (JCPDF #00-054-0752).

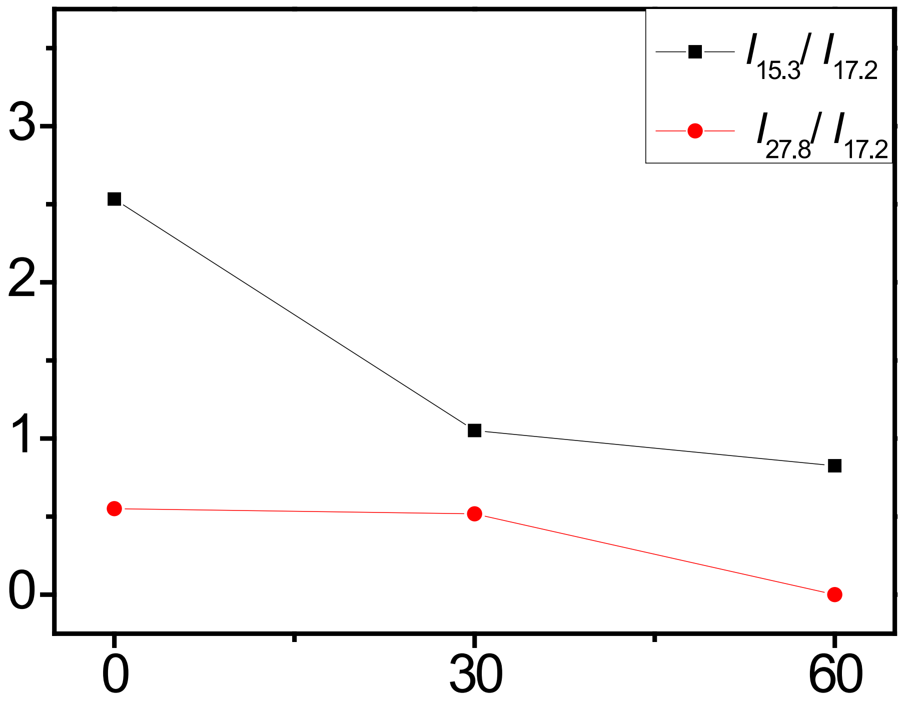

Supplementary Figure 26. The cubic $CsPbBr_3$ / hexagonal $NaYF_4$ ratio ($I_{15.3}/I_{17.2}$) and cubic $NaYF_4$ / hexagonal $NaYF_4$ ratio ($I_{27.8}/I_{17.2}$) derived from the XRD pattern of the heterostructured $CsPbBr_3$-$NaYF_4$:Yb,Tm hybrid nanocrystals in Figure 4 as a function of insulation time at 300 °C. The phase transition from cubic phase $NaYF_4$ to hexagonal phase $NaYF_4$ is confirmed.

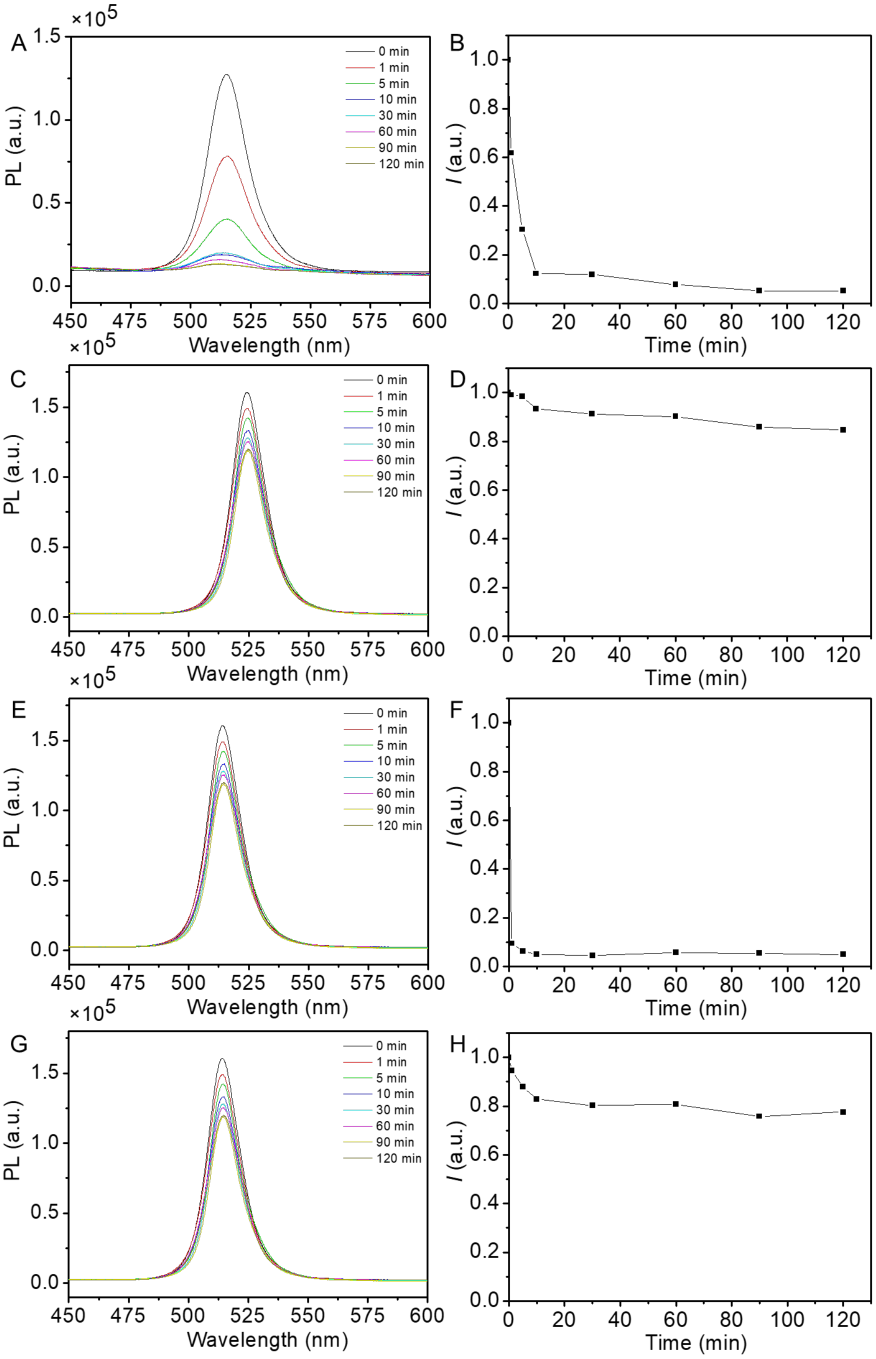

Supplementary Figure 27. PL intensity test was used to monitor the stabilities of $CsPbBr_3$ (A and B) and the heterostructure composite of $CsPbBr_3$-$NaYF_4$:Yb,Tm nanocrystals (C and D) in cyclohexane and ethanol (v/v = 9 : 1) mixed solvent. PL intensity test was used to monitor the stabilities of $CsPbBr_3$ (E and F) and the heterostructure composite of $CsPbBr_3$-$NaYF_4$:Yb,Tm nanocrystals (G and H) in cyclohexane and ethanol (v/v = 1 : 1) mixed solvent.

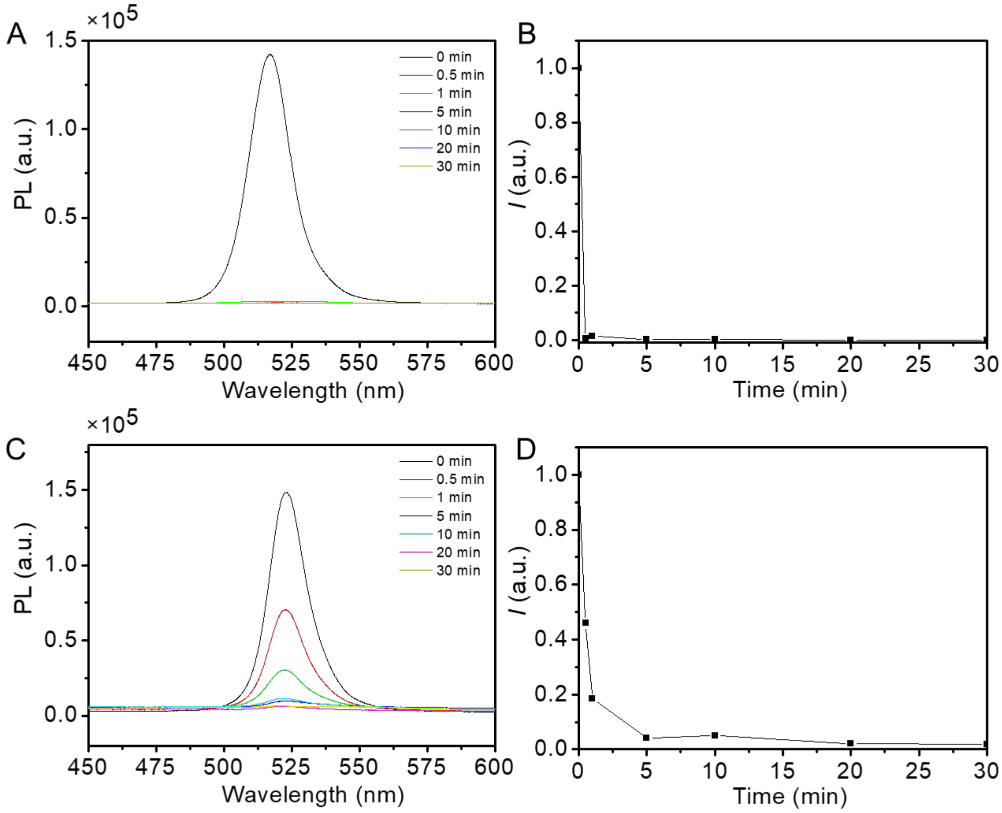

Supplementary Figure 28. PL intensity test was used to monitor the stabilities of $CsPbBr_3$ (A and B) and the heterostructure composite of $CsPbBr_3$-$NaYF_4$:Yb,Tm nanocrystals (C and D) in water solvent.

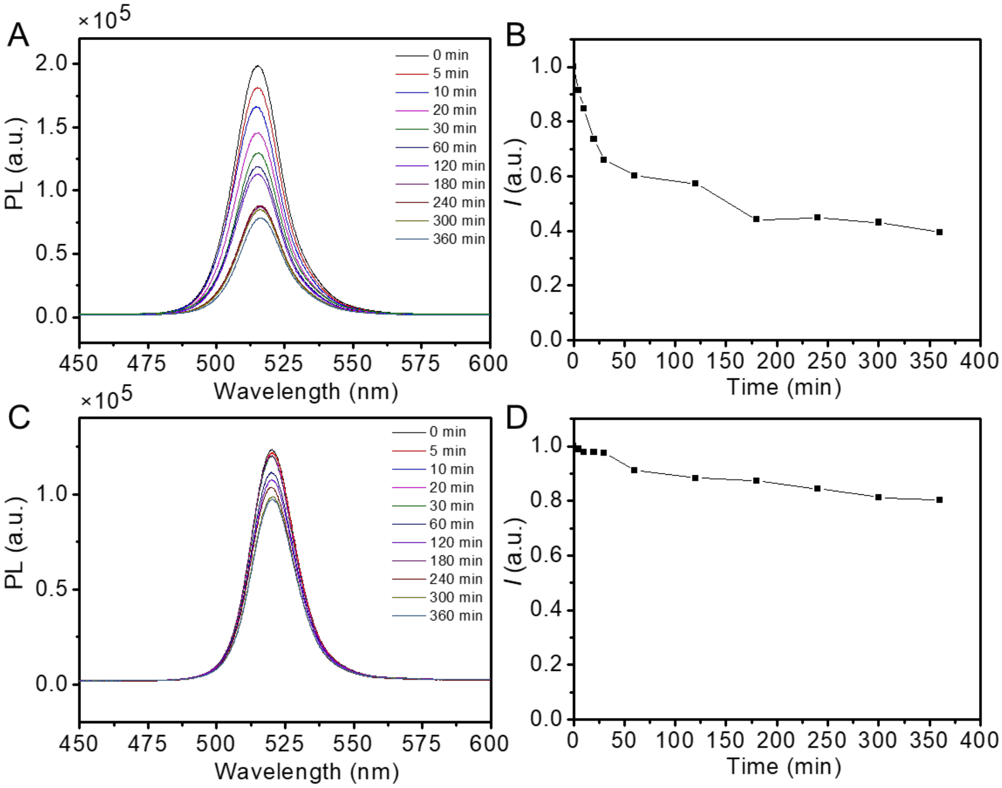

Supplementary Figure 29. PL intensity test was used to monitor the stabilities of $CsPbBr_3$ (A and B) and the heterostructure composite of $CsPbBr_3$-$NaYF_4$:Yb,Tm nanocrystals (C and D) under continuous ultraviolet light irradiation.

Supplementary Table 1. Exponential fitting results of $CsPbBr_3$ QDs.

Sample	$CsPbBr_3$
A_1	8153.3
τ_1 (ns)	20.6 (86%)
A_2	5662.1
τ_2 (ns)	4.7 (14%)
Average(ns)	18.4

Time-resolved fluorescence decay curve of $CsPbBr_3$ QDs was fitted by a double exponential (see eqs 1 and 2) function: $A(t)=A_0+A_1exp-(t-t_0)/\tau_1+A_2exp-(t-t_0)/\tau_2$ (eqs 1). The average lifetime was calculated using $\tau_{avg}=(A_1\tau_1^2+A_2\tau_2^2)/(A_1\tau_1+A_2\tau_2)$ (eqs 2).

Supplementary Table 2. Atomic ratios in the heterostructured $CsPbBr_3$-$NaYF_4$:Yb,Tm hybrid nanocrystals determined based on XPS measurement

**Element**	**Atomic%**
Cs3*d*	0.25
Pb4*f*	0.21
Br3*d*	0.78
F1*s*	65.96
Y3*d*	13.65
Na1*s*	14.78
Yb4*d*	3.10
Tm4*d*	1.27

Supplementary Table 3. PLQY of heterostructured nanocrystals before and after phase transition.

<table>
<tr><th>Before and after the phase transition</th><th>Samples</th><th colspan="2">Ex 980 nm-PLQY</th><th>Ex 365 nm-PLQY</th></tr>
<tr><td rowspan="4">Before the phase transition</td><td>Heterostructured $CsPbBr_3$-$NaYF_4$:Yb,Tm-150 ℃</td><td colspan="2">/</td><td>53%</td></tr>
<tr><td>Heterostructured $CsPbBr_3$-$NaYF_4$:Yb,Tm-200 ℃</td><td colspan="2">/</td><td>35%</td></tr>
<tr><td rowspan="2">Heterostructured $CsPbBr_3$-$NaYF_4$:Yb,Tm-250 ℃</td><td>UCNPs</td><td>0.02%</td><td rowspan="2">12%</td></tr>
<tr><td>$CsPbBr_3$</td><td>/</td></tr>
<tr><td rowspan="4">After the phase transition</td><td rowspan="2">Heterostructured $CsPbBr_3$-$NaYF_4$:Yb,Tm-300 ℃-0 min</td><td>UCNPs</td><td>0.139%</td><td rowspan="2">23%</td></tr>
<tr><td>$CsPbBr_3$</td><td>0.011%</td></tr>
<tr><td rowspan="2">Heterostructured $CsPbBr_3$-$NaYF_4$:Yb,Tm-300℃-60 min</td><td>UCNPs</td><td>0.223%</td><td rowspan="2">21%</td></tr>
<tr><td>$CsPbBr_3$</td><td>0.027%</td></tr>
</table>

Supplementary Table 4. PLQY of the $CsPbBr_3$ nanocrystals and heterostructured nanocrystals

Samples	Ex 365 nm-PLQY
Heterostructured $CsPbBr_3$-$NaYF_4$:Yb,Tm-150 ℃	53%
Heterostructured $CsPbBr_3$-$NaYF_4$:Yb,Tm-200 ℃	35%
Heterostructured $CsPbBr_3$-$NaYF_4$:Yb,Tm-250 ℃	12%
Heterostructured $CsPbBr_3$-$NaYF_4$:Yb,Tm-300 ℃-0 min	23%
Heterostructured $CsPbBr_3$-$NaYF_4$:Yb,Tm-300℃-60 min	21%
Conventional $CsPbBr_3$	65%
$CsPbBr_3$--300℃-60 min	0%

Supplementary Table 5. Exponential fitting results of $NaYF_4$:30%Yb,0.5%Tm nanocrystals, heterostructure composite of $CsPbBr_3$-$NaYF_4$:Yb,Tm nanocrystals and $CsPbBr_3$/ $NaYF_4$: 30%Yb,0.5%Tm mixture under 980 nm excitation (monitored at the emission of 478 nm).

Sample	$NaYF_4$:30%Yb,0.5%Tm	heterostructure composite of $CsPbBr_3$-$NaYF_4$:Yb,Tm	$CsPbBr_3$/ $NaYF_4$: 30%Yb,0.5%Tm mixture
A_1	5.25	1.05	2.54
τ1 (ns)	0.66×10^6 (100%)	0.71×106 (6.7%)	0.59×10^6 (100%)
A2		41.3	
τ2 (ns)		0.25×106 (93.3%)	
Average(ns)	0.66×10^6	0.29×10^6	0.59×10^6

Time-resolved fluorescence decay curves of $NaYF_4$:30%Yb,0.5%Tm nanocrystals and $CsPbBr_3$/ $NaYF_4$: 30%Yb,0.5%Tm mixture were fitted by a single exponential (see eqs 1 and 2) function: $A(t)=A_0+A_1\exp-(t-t_0)/\tau_1$ (eqs 1). The average lifetime was calculated using $\tau_{avg}=\tau_1$ (eqs 2). Time-resolved fluorescence decay curve of heterostructured $CsPbBr_3$-$NaYF_4$:Yb,Tm nanocrystals was fitted by a double exponential (see eqs 1 and 2) function: $A(t)=A_0+A_1\exp-(t-t_0)/\tau_1+A_2\exp-(t-t_0)/\tau_2$ (eqs 1). The average lifetime was calculated using $\tau_{avg}=(A_1\tau_1^2+A_2\tau_2^2)/(A_1\tau_1+A_2\tau_2)$ (eqs 2).

Supplementary Table 6. The FRET efficiency of of $NaYF_4$:30%Yb,0.5%Tm nanocrystals, heterostructure composite of $CsPbBr_3$-$NaYF_4$:Yb,Tm nanocrystals and $CsPbBr_3$/ $NaYF_4$: 30%Yb,0.5%Tm mixture under 980 nm excitation.

τ and Eff	$NaYF_4$:30%Yb,0.5%Tm	heterostructure composite of $CsPbBr_3$-$NaYF_4$:Yb,Tm	$CsPbBr_3$/ $NaYF_4$: 30%Yb,0.5%Tm mixture
τ_D (ns)	0.66×10^6	0.66×10^6	0.66×10^6
$\tau_{D\text{-}A}$ (ns)	/	0.29×10^6	0.59×10^6
$Eff = 1 - \tau_{D-A}/\tau_D$	/	56%	11%

Supplementary Table 7. Exponential fitting results of the $CsPbBr_3$ nanocrystals heterostructure composite of $CsPbBr_3$-$NaYF_4$:Yb,Tm nanocrystals under 365 nm, and 980 nm excitation respectively (reference taken from the 520 nm emission).

Sample	$CsPbBr_3$	heterostructure composite of $CsPbBr_3$-$NaYF_4$:Yb,Tm
A_1	8153.3	2233.06355
τ1 (ns)	20.6 (86%)	0.37×10^6 (100%)
A2	5662.1	
τ2 (ns)	4.7 (14%)	
Average(ns)	18.4	0.37×10^6

The time-resolved fluorescence decay curve of the $CsPbBr_3$ nanocrystals was fitted by a single exponential (see eqs 1 and 2) function: $A(t)=A_0+A_1exp-(t-t_0)/\tau_1+A_2exp-(t-t_0)/\tau_2$ (eqs 1). The average lifetime was calculated using $\tau_{avg}=(A_1\tau_1^2+A_2\tau_2^2)/(A_1\tau_1+A_2\tau_2)$ (eqs 2). The time-resolved fluorescence decay curve of the heterostructured $CsPbBr_3$-$NaYF_4$:Yb,Tm nanocrystals was fitted by a sigle exponential (see eqs 1 and 2) function: $A(t)=A_0+A_1exp-(t-t_0)/\tau_1$ (eqs 1). The average lifetime was calculated using $\tau_{avg}=\tau_1$ (eqs 2).

Supplementary Table 8. Exponential fitting results of $NaYF_4$:30%Yb,0.5%Tm nanocrystals and the heterostructure composite of $CsPbBr_3$-$NaYF_4$:Yb,Tm nanocrystals at different fold under 980 nm excitation (reference taken from the 478 nm emission).

Sample	$NaYF_4$:30%Yb ,0.5%Tm	heterostructure composite of $CsPbBr_3$-$NaYF_4$:Yb,Tm	0.2 fold heterostructure composite of $CsPbBr_3$-$NaYF_4$:Yb,Tm	5 fold heterostructure composite of $CsPbBr_3$-$NaYF_4$:Yb,Tm
A_1	5.25	1.05	7.99	66.8
τ1 (ns)	0.66×10^6 (100%)	0.71×10^6 (6.7%)	0.49×10^6 (100%)	0.25×10^6 (100%)
A2		41.3		
τ2 (ns)		0.25×10^6 (93.3%)		
Average(ns)	0.66×10^6	0.29×10^6	0.49×10^6	0.25×10^6

The time-resolved fluorescence decay curves of $NaYF_4$:30%Yb,0.5%Tm nanocrystals, 0.2 fold heterostructured $CsPbBr_3$-$NaYF_4$:Yb,Tm nanocrystals, and 5 fold heterostructured $CsPbBr_3$-$NaYF_4$:Yb,Tm nanocrystals were fitted by a single exponential (see eqs 1 and 2) function: $A(t)=A_0+A_1\exp-(t-t_0)/\tau_1$ (eqs 1). The average lifetime was calculated using $\tau_{avg}=\tau_1$ (eqs 2). The time-resolved fluorescence decay curve of the heterostructured $CsPbBr_3$-$NaYF_4$:Yb,Tm nanocrystals was fitted by a double exponential (see eqs 1 and 2) function: $A(t)=A_0+A_1\exp-(t-t_0)/\tau_1+A_2\exp-(t-t_0)/\tau_2$ (eqs 1). The average lifetime was calculated using $\tau_{avg}=(A_1\tau_1^2+A_2\tau_2^2)/(A_1\tau_1+A_2\tau_2)$ (eqs 2).

Supplementary Table 9. The FRET efficiency of $NaYF_4$:30%Yb,0.5%Tm nanocrystals and the heterostructure composite of $CsPbBr_3$-$NaYF_4$:Yb,Tm nanocrystals at different fold under 980 nm excitation.

τ and Eff	$NaYF_4$:30%Yb ,0.5%Tm	heterostructure composite of $CsPbBr_3$-$NaYF_4$:Yb,Tm	0.2 fold heterostructure composite of $CsPbBr_3$-$NaYF_4$:Yb,Tm	5 fold heterostructure composite of $CsPbBr_3$-$NaYF_4$:Yb,Tm
τ_D (ns)	0.66×10^6	0.66×10^6	0.66×10^6	0.66×10^6
$\tau_{D\text{-}A}$ (ns)	/	0.29×10^6	0.49×10^6	0.25×10^6
$Eff = 1 - \tau_{D-A}/\tau_D$	/	56%	25.8%	62.1%

Supplementary Table 10. Exponential fitting results of the heterostructured $CsPbBr_2/Cl_1$-$NaYF_4$:Yb,Tm nanocrystals and the heterostructured $CsPbBr_2/I_1$-$NaYF_4$:Yb,Tm nanocrystals under 980 nm excitation (reference taken from the 478 nm emission).

Sample	heterostructured $CsPbBr_2/Cl_1$-$NaYF_4$:Yb,Tm	heterostructured $CsPbBr_2/I_1$-$NaYF_4$:Yb,Tm
A_1	65653.7	14860.2
τ_1 (ns)	0.23×10^6 (100%)	0.48×10^6 (6.7%)
Average(ns)	0.23×10^6	0.48×10^6

The time-resolved fluorescence decay curve of the heterostructured $CsPbBr_2/Cl_1$-$NaYF_4$:Yb,Tm nanocrystals and the heterostructured $CsPbBr_2/I_1$-$NaYF_4$:Yb,Tm nanocrystals were fitted by a single exponential function (see eqs 1 and 2): $A(t)=A_0+A_1\exp-(t-t_0)/\tau_1$ (eqs 1). The average lifetime was calculated using $\tau_{avg}=\tau_1$ (eqs 2).
